# The *Solanum tuberosum KST1* partial promoter as a tool for guard cell expression in multiple plant species

**DOI:** 10.1093/jxb/erx159

**Published:** 2017-05-22

**Authors:** Gilor Kelly, Nitsan Lugassi, Eduard Belausov, Dalia Wolf, Belal Khamaisi, Danja Brandsma, Jayaram Kottapalli, Lena Fidel, Batsheva Ben-Zvi, Aiman Egbaria, Atiako Kwame Acheampong, Chuanlin Zheng, Etti Or, Assaf Distelfeld, Rakefet David-Schwartz, Nir Carmi, David Granot

**Affiliations:** 1 Institute of Plant Sciences, Agricultural Research Organization, The Volcani Center, Rishon LeZion, Israel; 2 Horticulture and Product Physiology, Wageningen University, AP Wageningen, The Netherlands; 3 Faculty of Life Sciences, Department of Molecular Biology and Ecology of Plants, Tel Aviv University, Israel

**Keywords:** Confocal imaging, GFP, guard cell-specific promoters, *KST1* promoter, stomata

## Abstract

To date, guard cell promoters have been examined in only a few species, primarily annual dicots. A partial segment of the potato (*Solanum tuberosum*) *KST1* promoter (*KST1* partial promoter, *KST1*_ppro_) has previously been shown to confer guard cell expression in potato, tomato (*Solanum lycopersicum*), citrus [Troyer citrange (*C. sinensis*×*Poncirus trifoliata*)], and Arabidopsis (*Arabidopsis thaliana*). Here, we describe an extensive analysis of the expression pattern of *KST1*_ppro_ in eight (previously reported, as well as new) species from five different angiosperm families, including the *Solanaceae* and the *Cucurbitaceae*, Arabidopsis, the monocot barley (*Hordeum vulgare*), and two perennial species: grapevine (*Vitis vinifera*) and citrus. Using confocal imaging and three-dimensional movies, we demonstrate that *KST1*_ppro_ drives guard cell expression in all of these species, making it the first dicot-originated guard cell promoter shown to be active in a monocot and the first promoter reported to confer guard cell expression in barley and cucumber (*Cucumis sativus*). The results presented here indicate that *KST1*_ppro_ can be used to drive constitutive guard cell expression in monocots and dicots and in both annual and perennial plants. In addition, we show that the *KST1*_ppro_ is active in guard cells shortly after the symmetric division of the guard mother cell and generates stable expression in mature guard cells. This allows us to follow the spatial and temporal distribution of stomata in cotyledons and true leaves.

## Teaser

The *KST1* partial promoter is expressed in guard cells immediately after guard mother cell division and drives constitutive guard cell expression in dicots and monocots, and under drought and ABA treatment.

## Introduction

Stomata, composed of two guard cells, are dynamic pores found in the epidermal layer of plants which balance the plant’s loss of water through transpiration with the uptake of CO_2_ for photosynthesis. As such, the mechanisms responsible for adjusting stomatal aperture in response to environmental and endogenous stimuli play a pivotal role throughout the life of the plant, shaping its development and physiology. Over the last three decades, great efforts have been made to elucidate the mechanisms that control stomatal behavior by modulating expression of various genes. Some of these studies were done with non-specific promoters such as the global 35S promoter, which may have had indirect effects. In light of that potential complication, the use of guard cell-specific promoters is an attractive option, and the isolation of such promoters has enabled the study of the role of specific genes directly in the context of guard cells, avoiding any indirect pleiotropic effects that might have arisen from expression in other cell types and tissues.

To date, several complete, partial, and synthetic promoters have been tested for guard cell expression using reporter genes such as β-glucuronidase (GUS) and green fluorescent protein (GFP). The promoters that drive expression in guard cells can be divided into two types based on their guard cell specificity (see Supplementary Table S1 at *JXB* online): type I promoters drive exclusive expression in guard cells and type II promoters drive preferred expression in guard cells with additional low expression in other vegetative tissues such as roots and veins. For example, the *MYB60*, *CYP86A2*, and *GC1* promoters are expressed exclusively in guard cells; whereas the *RHA1*, *PAO3*, *ROP11*, and *ROPGEF4* promoters display dominant-preferred expression in guard cells, but are also expressed in roots (Supplementary Table S1). Yet, only a limited number of studies have examined the expression of guard cell-specific promoters in various species, and those studies have mainly involved *Brassicaceae* and *Solanaceae* species (Hooker *et al.*, 2002; Yang *et al.*, 2008; Han *et al.*, 2013; Kelly *et al.*, 2013; Rusconi *et al.*, 2013). For example, the guard cell promoters *GC1*, *CER6*, and *TGG1* and the *SLSP* partial promoter were tested in Arabidopsis and tobacco (*Nicotiana tabacum*), the potato *AGPase* promoter was tested in potato, tobacco, and Arabidopsis, and the grapevine *SIRK* promoter was assayed in grapevine and Arabidopsis (Müller-Röber *et al.*, 1994, 1995; Berger and Altman, 2000; Hooker *et al.*, 2002; Husebye *et al.*, 2002; Pratelli *et al.*, 2002; Yang *et al.*, 2008; Han *et al.*, 2013). These studies suggest that the activity of guard cell-specific promoters might be universal. However, a recent study has pointed to a promoter limitation between dicots and monocots, as the *MYB60* promoter isolated from Arabidopsis was active in guard cells of *Solanaceae* species (tobacco and tomato), but failed to drive expression in rice (*Oryza sativa*; Rusconi *et al.*, 2013).

The potato *StKST1* gene encodes a guard cell potassium (K^+^) influx channel necessary for stomatal opening, and its promoter drives expression of GUS in potato guard cells and flowers (Plesch *et al.*, 2001). Deletion analysis of the *KST1* promoter identified a minimal *KST1* promoter of 642 bp (*KST1*_ppro_). When that minimal promoter was used, expression in flowers was abolished, while expression in guard cells remained high (Plesch *et al.*, 2001). Recent studies have suggested that *KST1*_ppro_ drives exclusive expression in guard cells of Arabidopsis and tomato (Kelly *et al.*, 2013; Sade *et al.*, 2014). In addition to the function of the *KST1* promoter in the guard cells of potato, tomato, and Arabidopsis (Plesch *et al.*, 2001; Kelly *et al.*, 2013; Sade *et al.*, 2014), it was also discovered that, in citrus, *KST1*_ppro_ drives dominant-preferred expression in guard cells with additional low-level expression in epidermal cells (Lugassi *et al.*, 2015). In tobacco, the full-length *KST1* promoter was shown to be active in guard cells, but that analysis was limited to epidermal peels and did not include the examination of other tissues (Muller-Rober *et al.*, 1998). Though *KST1*_ppro_ seems to be a promising tool for studying gene expression in guard cells, its use has so far been quite limited (Plesch *et al.*, 2001; Kelly *et al.*, 2013; Sade *et al.*, 2014; Lugassi *et al.*, 2015; Antunes *et al.*, 2017). In this study, we summarize the current knowledge regarding guard cell promoters and list them as type I and type II promoters (Supplementary Table S1). We focus only on promoters that are active in guard cells and describe the species assayed and the verification method used. For type II promoters, we state the expression pattern (other than guard cells). In addition, we analyzed the *KST1*_ppro_ expression pattern in eight species, including a thorough re-examination of the previously described *KST1*_ppro_*::GFP* (GCGFP) species (tomato, Arabidopsis, and citrus), as well as newly introduced GCGFP species (tobacco, cucumber, grapevine, and barley). Expression of *KST1*_ppro_ in potato was re-analyzed here as well, using GFP instead of the previously described *KST1*_ppro_*::GUS* (Plesch *et al.*, 2001). The results of this work indicate that the *KST1*_ppro_ may be used as a universal tool for achieving guard cell expression. Moreover, this is the first guard cell promoter reported to be active in cucumber and barley. The specific expression of *KST1*_ppro_ in newly formed guard cells allowed us to track the spatial distribution of stomata in cotyledons and true leaves over time, starting from the early stages of germination and seedling development.

## Materials and methods

### Plant material and growth conditions

Plant material used in this study included Arabidopsis (Columbia, Col-0), potato (*S. tuberosum* L. cv. Desirée), tobacco (*N. tabacum* cv. *Samsun NN*), cucumber (*C. sativus* cv. Ilan, Syngenta), grapevine (*V. vinifera* cv. Sugarone), barley (*H. vulgare* cv. Golden Promise), citrus (*C. sinensis*×*Poncirus trifoliata*), and tomato (*S. lycopersicum* cv. MP-1) plants. Arabidopsis plants were grown either on half-strength Murashige and Skoog medium (1/2 MS; Duchefa Biochemie, The Netherlands) agar plates or in soil. The soil in which the Arabidopsis and citrus plants were grown contained (w/w) 30% vermiculite, 30% peat, 20% tuff, and 20% perlite (Shaham-Ada, Israel). Tomato, tobacco, cucumber, grapevine, citrus, and barley plants were grown in a mixture of 70% tuff and 30% peat (Shaham-Ada), and potato plants were grown in a mixture of peat, quartz, and coconut fibers (Green 90, Even Ari, Israel). Tomato, potato, tobacco, cucumber, barley, and citrus plants were grown in a temperature-controlled greenhouse under natural conditions. The Arabidopsis and grapevine plants were grown in growth rooms kept at 22 °C, with a 16 h light/8 h dark photoperiod.

### Generation of transgenic plants

All plant transformations in this study (with the exception of those of barley) were performed using *Agrobacterium tumefaciens* strain EHA105 harboring the kanamycin-resistant *pGreen* binary vector containing the *KST*_ppro_*::GFP* segment and *pSoup* as helper plasmid. For a detailed description of the barley transformation, see below. The Arabidopsis, tomato, and citrus plants expressing GFP in their guard cells have been described previously (Kelly *et al.*, 2013; Lugassi *et al.*, 2015). Transformations of potato, tobacco, cucumber, grape, and barley were conducted as described below.

#### Potato transformation

Potato transformation was conducted according to the protocol described in Ginzberg *et al.* (2012) with minor modifications. Sterile potato leaf discs were incubated with *Agrobacterium* for 5–10 min and then shifted to MS medium containing 3% sucrose (Suc; Duchefa), 200 µM acetosyringone (AS; Sigma-Aldrich, Israel) for 2 d in the dark. Explants were then transferred to MS+3% Suc with 0.1 mg l^–1^ 6-benzylaminopurine (BA; Sigma-Aldrich), 5 mg l^–1^ naphthalene acetic acid (NAA; Duchefa) supplemented with 500 mg l^–1^ Claforan (Cla; Cefotaxim, Duchefa) and 50 mg l^–1^ kanamycin (Kan; Duchefa). Plates were incubated (25 °C, 16/8 h light/dark photoperiod) for 10 d. Then, explants were shifted to selection medium [MS, 3% Suc, 2 mg l^–1^ zeatin riboside (Duchefa), 0.02 mg l^–1^ gibberellic acid (GA_3_; Duchefa), 0.02 mg l^–1^ NAA, 500 mg l^–1^ Cla, and 50 mg l^–1^ Kan]. After ~6 weeks, plantlets were transferred to rooting medium (MS, 3% Suc, 500 mg l^–1^ Cla and 50 mg l^–1^ Kan). Rooted plantlets were transferred to soil and were kept for a 10 d hardening before they were transferred to a greenhouse.

#### Tobacco transformation

Tobacco was transformed using *Agrobacterium*-mediated transformation (Horsch *et al.*, 1985; Gallois and Marinho, 1995). Leaf discs of sterile leaves were placed upside down in induction medium (MS, 3% Suc, 1 mg l^–1^ BA, 2 mg l^–1^ NAA, and 100 µM AS) for 24 h at 25 °C in the dark. Leaf discs were then immersed with *Agrobacterium* for 2 min, dried, and transferred back to induction medium for another 2–3 d. Explants were then shifted to selection medium (MS containing 1 mg l^–1^ BA, 0.1 mg l^–1^ NAA, 500 mg l^–1^ Cla, and 200 mg l^–1^ Kan) for selection. Small plantlets appeared after ~1 month and were shifted to MS medium containing 0.1 mg l^–1^ BA, 500 mg l^–1^ Cla, and 200 mg l^–1^ Kan. Developed plantlets (2 cm long) were transferred to rooting medium (MS+500 mg l^–1^ Cla, and 200 mg l^–1^ Kan). Rooted plantlets were transferred to soil and were kept for a 10 d hardening period before they were transferred to the greenhouse.

#### Cucumber transformation

Cucumber was transformed using the *Agrobacterium*-mediated transformation precisely as described by Gal-On *et al.* (2005).

#### Grape transformation

To generate transgenic grapevine lines, the *KST1*_ppro_*::GFP* construct was transformed by *Agrobacterium* into embryonic calli as previously described (Perl *et al.*, 2004).

#### Barley transformation

*Agrobacterium*-mediated transformation of immature embryos of barley was performed following the protocol of Harwood *et al.* (2009). A *pBRACT* vector provided by the John Innes Centre (Norwich, UK) and containing the *KST1*_ppro_*::GFP* segment was used with the *pSoup* helper and the *Agrobacterium* strain AGL1.

### Characterization of transformants

Following the screening on Kan selection media, PCR was used to distinguish between transgenic and non-transgenic plants. The primers used for amplification were as follows: KST_F, TCTCAACAAATTCCCCTTGC; KST_R, GGGTGATACA CGGGTCAAGT; GFP_R, TGCTCAGGTAGTGGTTGTCG; GFP_F, ACGTAAACGGCCACAAGTTC; nptII_F, CACGCAGG TTCTCCGGCCGC; and nptII_R, TGCGCTGCGAATCGGGA GCG. These primers were designed to amplify *KST*_ppro_ (KST_F/R), GFP (GFP_F/R), *KST1*_ppro_ together with GFP (KST_F/GFP_R), and the selectable marker neomycin phosphotransferase II (nptII_F/R). Positive plants were then taken for confocal microscope analysis (see below) in which GFP fluorescence was verified. A similar fluorescence pattern was observed for all of the positive transgenic lines tested for each species.

### Promoter activity in response to drought and abscisic acid (ABA)

For the drought experiment of GCGFP tomato plants, leaflets were detached and dried under greenhouse conditions for 4 h until wilting was visible and then analyzed. For the Arabidopsis drought experiment, irrigation was stopped for 6 d. We found that growth was delayed during this period. For ABA experiments, Arabidopsis GCGFP leaves were excised and immediately immersed (petiole-deep) in artificial xylem sap solution containing 1 mM KH_2_PO_4_, 1 mM K_2_HPO_4_, 1 mM CaCl_2_, 0.1 mM MgSO_4_, 3 mM KNO_3_, and 0.1 mM MnSO_4_, pH 5.8 with HCl (AXS; Wilkinson *et al.*, 1998) or AXS supplemented with 10 μM ABA for 30 min, according to a previously described procedure (Shatil-Cohen *et al.*, 2011). Following treatment, samples were taken for RNA extraction and confocal microscopy imaging.

### Confocal microscopy imaging

Images were acquired using the OLYMPUS IX 81 (Japan) inverted laser scanning confocal microscope (FLUOVIEW 500) equipped with a 488 nm argon ion laser and a 60 × 1.0 NA PlanApo water immersion objective. GFP was excited by 488 nm light and the emission was collected using a BA 505–525 filter. A BA 660 IF emission filter was used to observe chlorophyll autofluorescence. Confocal optical sections were obtained at 0.5 µm increments. The images were color coded green for GFP and magenta for chlorophyll autofluorescence. To evaluate the intensity of GFP fluorescence, images were analyzed using the ImageJ (http://rsb.info.nih.gov/ij/) software histogram tool. 3-D images and movies were obtained using the FLUOVIEW 500 supplied with the confocal laser scanning microscope.

### Quantitative real-time PCR analysis

RNA extraction, cDNA preparation, and quantitative real-time PCR analysis were performed precisely as described by Lugassi *et al.* (2015). Data were normalized using Arabidopsis *TUB2* (AT5G62690) or tomato *SlCyP* (cyclophilin accession; M55019) as reference genes. The primers used for amplification are specified in Supplementary Table S2.

## Results

### *Expression pattern of the* KST1 *partial promoter*

**Fig. 1.**
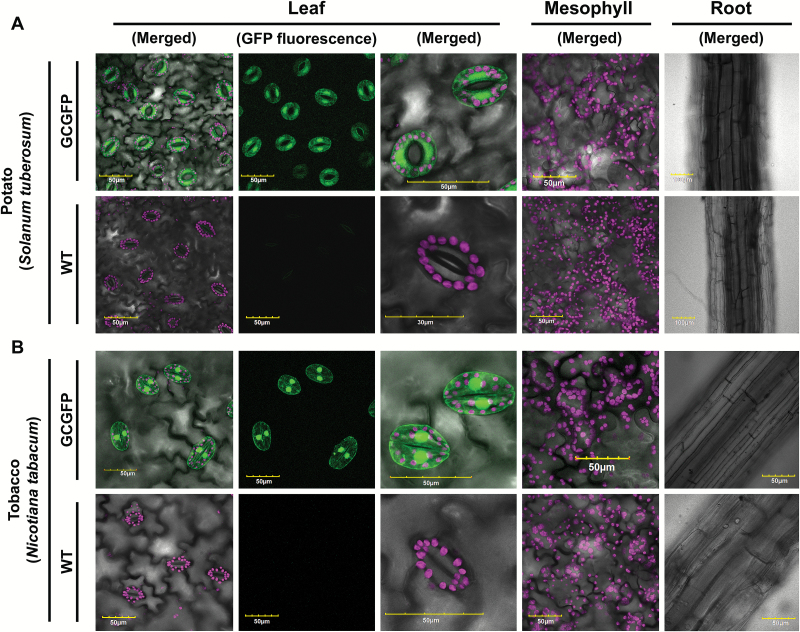
GFP expression under the control of *KST1*_ppro_ is specific to guard cells in potato (A) and tobacco (B). (A, B) Confocal images of leaf, mesophyll, and root of wild-type (WT) and transgenic plants expressing GFP under the control of *KST1*_ppro_ (GCGFP). Unless mentioned otherwise, all panels are merged images of white light, chlorophyll autofluorescence (stained magenta), and GFP fluorescence (stained green). Scale bars (yellow) are defined in each image.

**Fig. 2.**
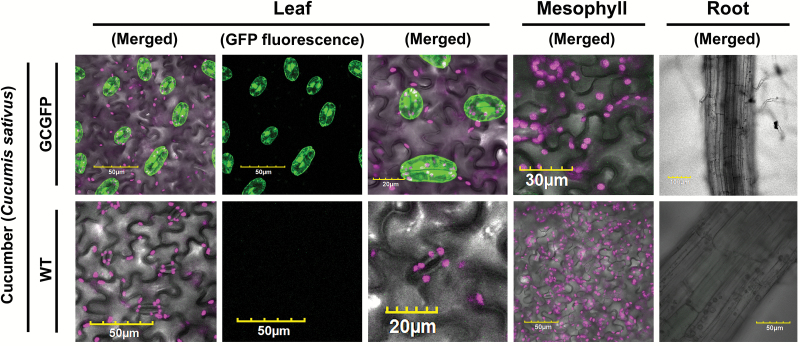
GFP expression under the control of *KST1*_ppro_ is specific to the guard cells of cucumber. Confocal images of leaf, mesophyll, and root of wild-type (WT) and transgenic plants expressing GFP under the control of *KST1*_ppro_ (GCGFP). Unless mentioned otherwise, all panels are merged images of white light, chlorophyll autofluorescence (stained magenta), and GFP fluorescence (stained green). Scale bars (yellow) are defined in each image.

**Fig. 3.**
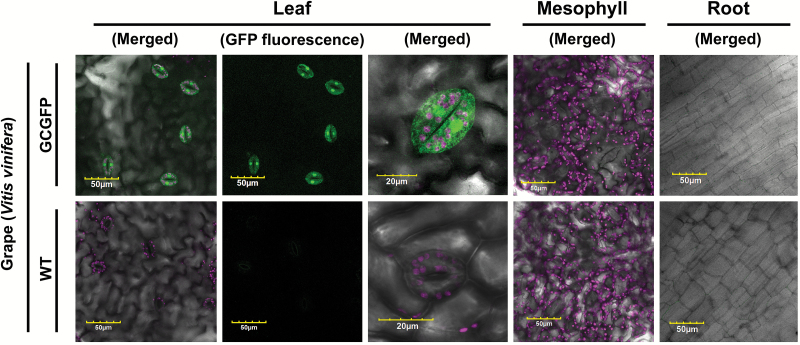
GFP expression under the control of *KST1*_ppro_ is specific to the guard cells of grapevine. Confocal images of leaf, mesophyll, and root of wild-type (WT) and transgenic plants expressing GFP under the control of *KST1*_ppro_ (GCGFP). Unless mentioned otherwise, all panels are merged images of white light, chlorophyll autofluorescence (stained magenta), and GFP fluorescence (stained green). Scale bars (yellow) are defined in each image.

**Fig. 4.**
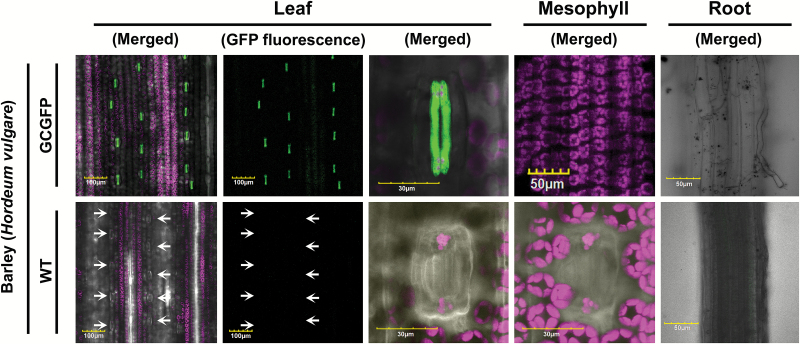
GFP under the control of *KST1*_ppro_ is specifically expressed in the guard cells of mature barley leaves. Confocal images of mature leaf, mesophyll, and root of wild-type (WT) and transgenic plants expressing GFP under the control of *KST1*_ppro_ (GCGFP). Unless mentioned otherwise, all panels are merged images of white light, chlorophyll autofluorescence (stained magenta), and GFP fluorescence (stained green). White arrows indicate the location of the stomata. Scale bars (yellow) are defined in each image.

**Fig. 5.**
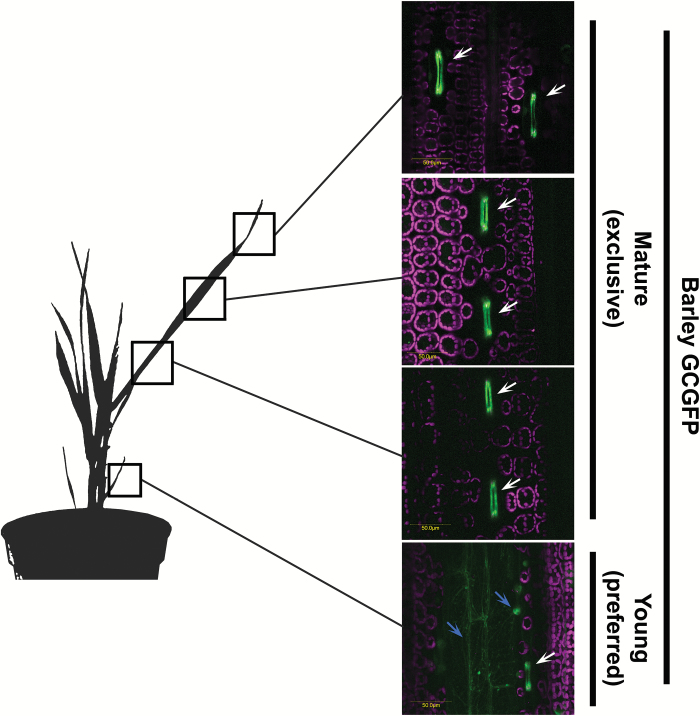
GFP under the control of *KST1*_ppro_ is preferentially or specifically expressed in the guard cells of young and mature barley leaves, respectively. Confocal images of young and mature leaves of transgenic plants expressing GFP under the control of *KST1*_ppro_ (GCGFP). All panels are merged images of white light, chlorophyll autofluorescence (stained magenta), and GFP fluorescence (stained green). White arrows indicate the location of the stomata. Blue arrows indicate the location of epidermal cells. Black squares indicate the location of the sample taken for imaging (a young leaf was taken from a newly developed tiller). Scale bars (yellow) are defined in each image.

*KST1*_ppro_ was originally tested in potato using GUS as a reporter gene (Plesch *et al.*, 2001) and was later examined using GFP as a reporter gene in Arabidopsis, tomato, and citrus (Kelly *et al.*, 2013; Sade *et al.*, 2014; Lugassi *et al.*, 2015). To study its expression in other species, we generated transgenic lines expressing GFP under the control of the *KST1*_ppro_ promoters of four additional species, tobacco, cucumber, grape, and barley, as well as potato lines expressing GFP under *KST1*_ppro_. We use the term GCGFP (an abbreviation of guard cell GFP) to refer to these lines. We also created potato GCGFP to compare with the previously described *KST1*_ppro_:*GUS* plants (Plesch *et al.*, 2001). Three to ten independent transgenic lines were assayed for each of the newly introduced GCGFP species, and we conducted an in-depth search for the presence of GFP fluorescence using a sensitive confocal microscope. The independent lines of each species had the same pattern of expression. The data were displayed as standard images (Figs 1–5; Supplementary Figs S1–S3) or as a 3-D movie that provides a 360° tour within the leaf including the epidermis and mesophyll (Supplementary Video S1). The combined data (still images and the movie) enabled us to identify the tissues and cell types in which *KST1*_ppro_ is expressed, with a high degree of confidence.

In agreement with the results of Plesch *et al.* (2001) who used GUS expression, we found in our work with GFP that in potato *KST1*_ppro_ drives guard cell-specific expression, with no expression in roots or mesophyll cells (Fig. 1A; Supplementary Fig. S1; Supplementary Video S1).

In previous work with tobacco, the expression pattern of the full-length *KST1* promoter was tested only in epidermal peels (Muller-Rober *et al.*, 1998). In the current study, the expression of the partial promoter in various vegetative tobacco tissues was analyzed. We found that *KST1*_ppro_ is expressed specifically in tobacco guard cells (Fig. 1B; Supplementary Fig. S1; Supplementary Video S1).

Cucumber GCGFP plants also display guard cell-specific expression and GFP fluorescence only in their guard cells (Fig. 2; Supplementary Fig. S1; Supplementary Video S1). To the best of our knowledge, this is the first guard cell promoter reported to be active in a *Cucurbitaceae* species. A similar guard cell-specific expression pattern was observed following thorough examination of tomato and Arabidopsis (Supplementary Fig. S2; Supplementary Video S1).

We have previously reported strong expression of GCGFP in the guard cells of perennial citrus plants (Lugassi *et al.*, 2015; Supplementary Fig. S3; Supplementary Video S1). In those plants, low expression was detected in epidermal cells of mature leaves (blue arrows in Supplementary Fig. S3), but not in young leaves. Nonetheless, GFP was not detected in the mesophyll cells or roots of the citrus plants (Lugassi *et al.*, 2015; Supplementary Fig. S3). In an effort to examine expression in another perennial species, we also examined *KST1*_ppro_ activity in grapevine (Fig. 3; Supplementary Fig. S1; Supplementary Video S1). GFP was detected only in guard cells and not in any vegetative, non-stomatal tissues such as vascular tissues, mesophyll, epidermis, or roots (Fig. 3; Supplementary Fig. S1). Two type I guard cell promoters were previously isolated from grapevine: *MYB60* and the stomatal inward rectifying K^+^ channel (SIRK; Pratelli *et al.*, 2002; Galbiati *et al.*, 2011). Yet, unlike *KST1*_ppro_, when the *SIRK* promoter was examined in Arabidopsis it did not retain its guard cell specificity, displaying expression in the xylem as well (Pratelli *et al.*, 2002).

We extended our assay to monocots and analyzed GCGFP barley plants expressing GFP under the *KST1*_ppro_ promoter (Fig. 4; Supplementary Fig. S1; Supplementary Video S1). In young leaves taken from newly developed tillers, GFP expression was detected in guard cells as well as epidermal cells (blue arrow in Fig. 5). However, once the leaves grew bigger, expression was detected only in guard cells (Fig. 5). Furthermore, the expression in the guard cells was uniform along the leaf (i.e. at the base, middle, and tip of the leaves), as shown in Fig. 5. Expression was not detected in subsidiary cells adjacent to the guard cells, in young or mature leaves. In a recent study, Arabidopsis *MYB60*, a type I promoter in Arabidopsis, tobacco, and tomato, failed to drive expression in rice (Rusconi *et al.*, 2013). To the best of our knowledge, the *KST1*_ppro_ promoter is the first dicot guard cell promoter found to be active in monocots.

### KST1*_ppro_ activity in response to drought and ABA*

**Fig. 6.**
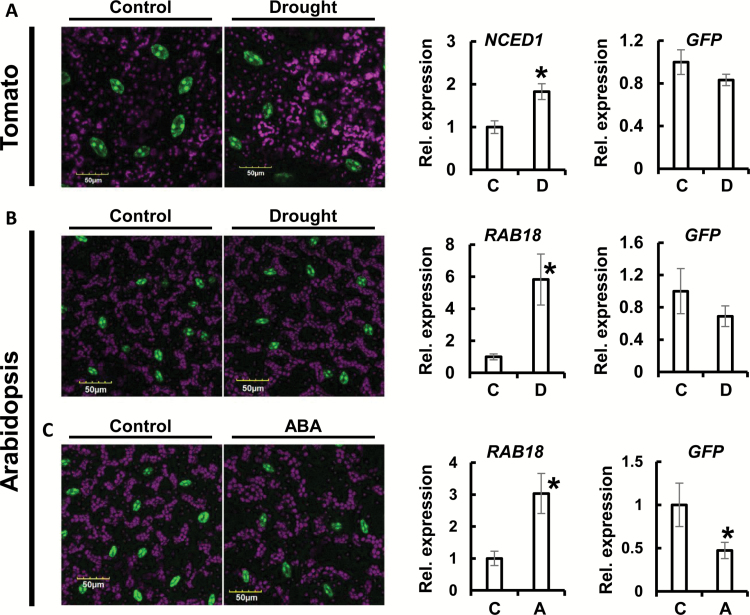
Confocal images and relative expression of GFP and ABA-related genes of GCGFP tomato and Arabidopsis plants in response to drought and application of ABA. (A) The left hand panels show tomato GCGFP leaflets 4 h after leaf detachment. (B) Arabidopsis GCGFP leaves from soil-grown plants after 6 d without irrigation. (C) Arabidopsis GCGFP leaves immersed (petiole-deep) in control solution or in 10 μM ABA for 30 min. Confocal images are merges of chlorophyll autofluorescence (stained magenta), and GFP fluorescence (stained green). Scale bars (yellow) are defined in each image. In the right-hand panels, relative expression of GFP, *RAB18*, and *NCED1* was determined using RNA extracted from control and treated leaves of the GCGFP plants used in the experiments shown in the left-hand panels. *TUB2* and *SlCyP* were used for normalization of the expression in Arabidopsis and tomato, respectively, and the expression of the control plants was set to 1. Data are means of five independent biological repeats ±SE. An asterisk denotes a significant difference (*t*-test, *P*<0.05). C, control; D, drought; A, ABA.

The activity of the *KST1*_ppro_ promoter under drought and ABA treatment was assayed in tomato and Arabidopsis GCGFP plants (Fig. 6). Detached tomato GCGFP leaflets were maintained under greenhouse conditions for 4 h to impose dehydration, until wilting was visible. The expression of the 9-*cis*-epoxycarotenoid dioxygenase (*SlNCED1*), a key enzyme in the biosynthesis of ABA (Nambara and Marion-Poll, 2005), indicated that the stress was active at that point (Fig. 6A). In spite of a slight, insignificant reduction, the GFP expression level remained unchanged, in line with the GFP fluorescence, which also remained similar to that of the fully turgid control leaflets (Fig. 6A). Similar results were obtained when Arabidopsis GCGFP plants were exposed to drought by stopping the irrigation for 6 d (Fig. 6B). Plants exposed to this drought treatment displayed delayed growth and up-regulation of the ABA-related gene *RAB18* (Lång and Palva, 1992), indicating that they experienced stress at this stage (Fig. 6B). The GFP fluorescence and GFP expression levels of the drought-treated Arabidopsis plants were similar to those of the fully irrigated control plants (Fig. 6B), just as observed for tomato. Taken together, these results demonstrate that the *KST1*_ppro_ promoter remains active under drought conditions.

In addition to the drought treatments, we also assayed *KST1*_ppro_ activity in response to treatment with ABA (Fig. 6C). Within 30 min of the application of a 10 μM ABA solution (petiole-deep application), there was an ~50% reduction in the expression of GFP together with a decline in GFP fluorescence, indicating the responsiveness of the promoter to ABA (Fig. 6C).

### *Use of* KST1 _ppro _::GFP *to track the spatial and temporal distribution of stomata in germinating Arabidopsis seedlings*

**Fig. 7.**
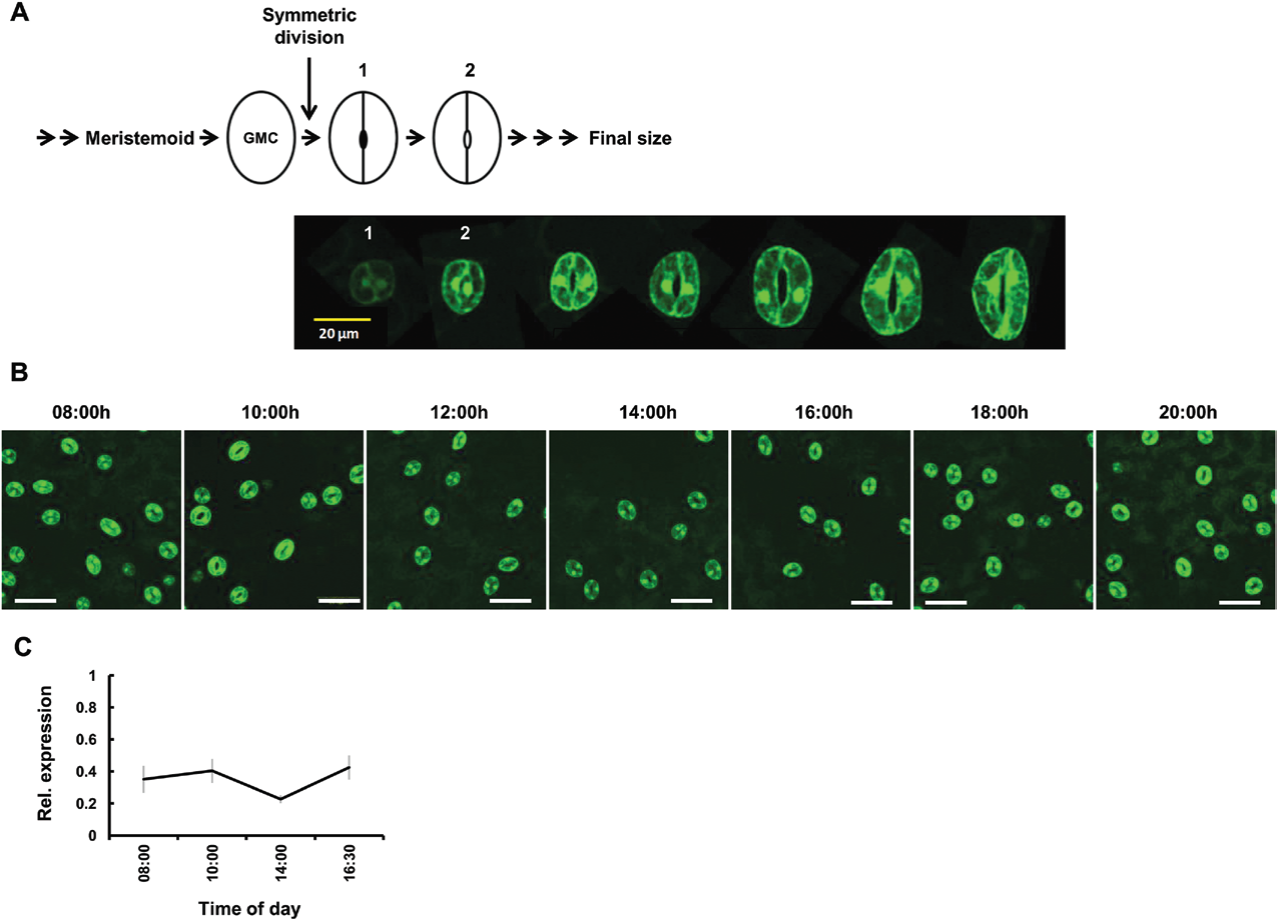
*KST1*_ppro_ expression during stomatal development and in mature leaves. (A) *KST1*_ppro_ expression during stomatal development. A schematic presentation of the developmental stages is shown at the top of the figure, from the guard mother cell (GMC), through its symmetric division; (1) guard cell formation immediately after the symmetric division of GMC (2) young stomata. Lower panel: confocal images of guard cells from cotyledons of Arabidopsis GCGFP plants. Stages 1 and 2 match the stages schematically described at the top of this figure. *KST1*_ppro_ is expressed in the newly formed guard cell (stage 1) and its expression increases and stabilizes in more mature guard cells. Expression is not detected in the GMC. (B) Promoter activity of *KST1*_ppro_ over the course of the day in mature Arabidopsis GCGFP leaves. Confocal images were taken at seven different time points over the course of a day; scale bar=50 µm. (C) Relative expression of GFP was determined using RNA extracted from developed leaves of the GCGFP plants used in (B) at four different points in time over the course of the day. *TUB2* was used for normalization. Data are means of five independent biological repeats ±SE. In (A) and (B) GFP fluorescence is stained green.

In the final stages of stomatal development, meristemoids are formed and undergo two additional steps to produce stomatal guard cells (Lau and Bergmann, 2012; Dow and Bergmann, 2014; Matos *et al.*, 2014). A meristemoid yields a guard mother cell (GMC) and the GMC undergoes a second step of symmetric division to form two young guard cells that mature and form an active stoma (Lau and Bergmann, 2012; Dow and Bergmann, 2014; Matos *et al.*, 2014). We used GCGFP Arabidopsis seedlings to follow the expression of *KST1*_ppro_ during stomatal formation (Fig. 7A). We found that *KST1*_ppro_ is expressed in newly formed Arabidopsis guard cells immediately after the symmetric division of a GMC and is not expressed in meristemoids or GMCs (Fig. 7A). The expression of *KST1*_ppro_ intensifies and stabilizes in more developed guard cells so that all mature stomata display strong GFP fluorescence (Fig. 7A). In addition, *KST1*_ppro_ seems to drive fairly steady expression throughout the day, as indicated by GFP intensity and measurements of GFP expression (Fig. 7B, C).

**Fig. 8.**
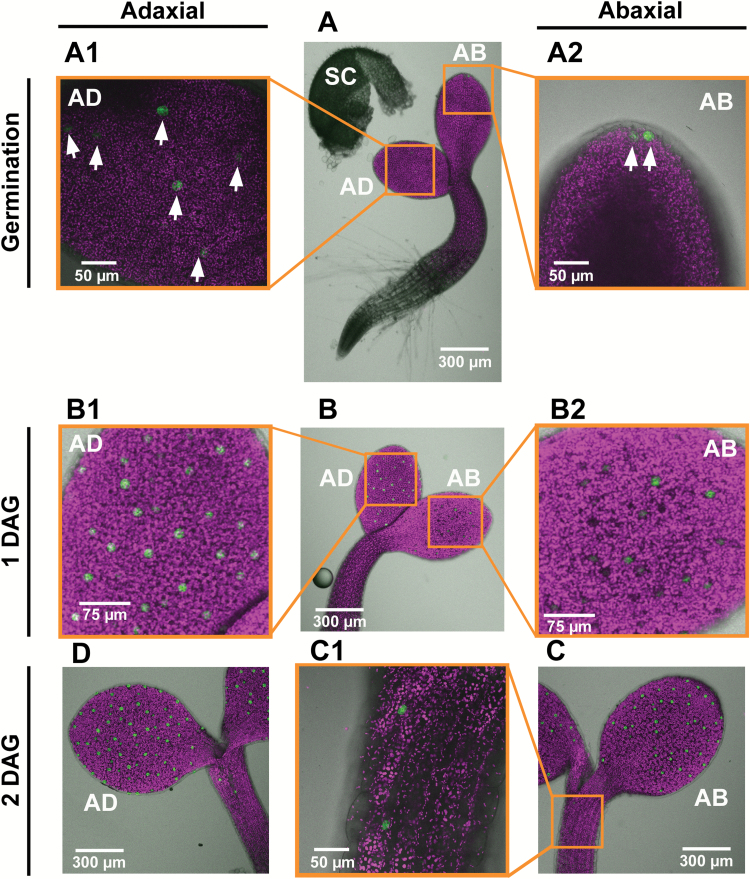
Guard cell formation in Arabidopsis GCGFP seedlings from germination through 2 days after germination (DAG). (A) Seedling at germination, immediately after the removal of the seed coat. At the germination stage, stomata are already visible on the adaxial and abaxial sides of the cotyledons. (A1) Enlargement of the adaxial (AD) side of the cotyledon. (A2) Enlargement of the abaxial (AB) side. The white arrows point to newly formed guard cells. (B) Seedling at 1 DAG. (B1) Enlargement of the adaxial side. (B2) Enlargement of the abaxial side. (C) Seedling at 2 DAG after germination. (C1) Enlargement of the hypocotyl in (C). (D) Adaxial side of seedling at 2 DAG. All panels are merged images of white light, chlorophyll autofluorescence (stained magenta), and GFP fluorescence (stained green). SC, seed coat.

**Fig. 9.**
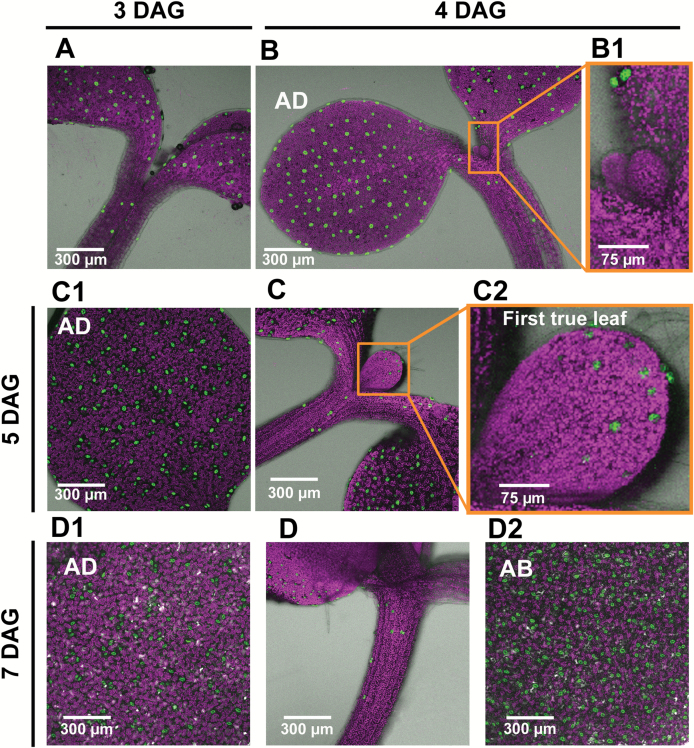
Guard cell formation in Arabidopsis GCGFP seedlings 3–7 days after germination (DAG). (A) Seedling at 3 DAG. (B) Seedling at 4 DAG. (B1) Enlargement of the first two true leaves shown in (B). (C) Seedling at 5 DAG. (C1) Adaxial side of cotyledon at 5 DAG. (C2) Enlargement of the first true leaf shown in (C). (D) Hypocotyl at 7 DAG. (D1) Adaxial side of the cotyledon of the seedling shown in (D). (D2) Abaxial side of the cotyledon of the seedling shown in (D). All panels are merged images of white light, chlorophyll autofluorescence (stained magenta), and GFP fluorescence (stained green). AD, adaxial; AB, abaxial.

**Fig. 10.**
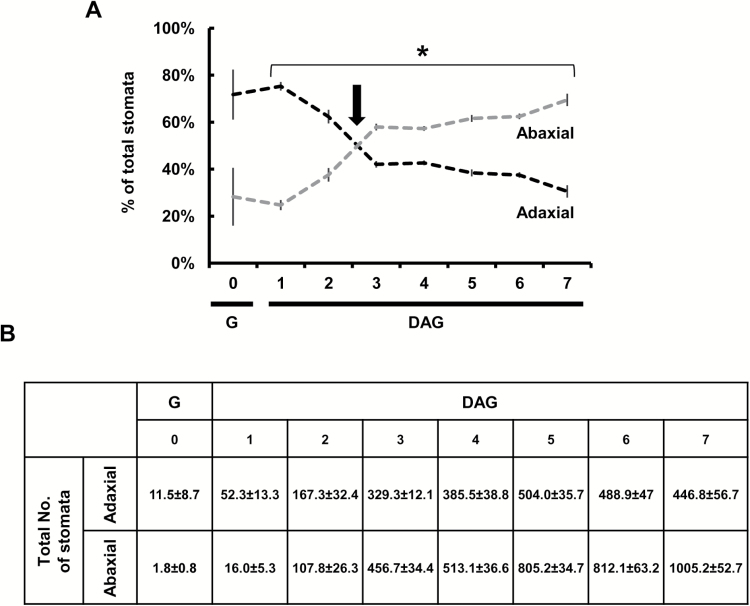
The spatio-temporal distribution of stomata in cotyledons of developing Arabidopsis seedlings including the distribution of the stomata between the abaxial and adaxial sides of cotyledons. (A) The total number of stomata (abaxial+adaxial sides of both cotyledons) was set to 100% and the percentage of stomata on the adaxial or abaxial side was documented from germination to 7 days after germination (DAG). The black arrow indicates the estimated inversion point, between 2 and 3 DAG. (B) The total number of stomata on the abaxial and adaxial surfaces of cotyledons was documented from germination to 7 DAG. (A, B) The total number of stomata was averaged each day for the adaxial (black dashed line) or abaxial (gray dashed line) sides of four independent seedlings, two cotyledons each ±SE. When not seen, the SE is smaller than the symbol. G, germination. Germination was defined as the time at which the seedling penetrated the seed coat. The asterisk denotes a significant difference (*t*-test, *P*<0.01).

The immediate and constitutive expression of *KST1*_ppro_ in newly formed guard cells allowed us to follow the timing of the appearance of stomata and their distribution across cotyledons and true leaves, using intact germinating Arabidopsis and tobacco seedlings. For this purpose, Arabidopsis seeds were sown on 1/2 MS agar plates and kept for 2 d at 4 °C and then transferred to a growth chamber at 22 °C with a 16 h light/8 h dark photoperiod. Germination was defined as the time at which the seedling penetrated the seed coat, ~24 h after the transfer to the growth chamber. Imaging of the intact seedling allowed us to follow the spatial and temporal distribution of stomata on the adaxial and the abaxial sides of the cotyledons of the same seedling simultaneously, up to 7 d after germination (Figs 8–10). It appears that stomata are formed first at the center of the adaxial side of the cotyledons of germinating seedlings and at the most distal end (the tip) of the abaxial side (Fig. 8A, A1, A2). More stomata are formed on the adaxial side than on the abaxial side during the first 2 d after germination (Figs 8, 10) and stomata on the adaxial side appear slightly more developed at 1 d after germination, as compared with those on the abaxial side, with more intense GFP fluorescence and clear pore formation (Fig. 8B, B1, B2). The number of stomata on the adaxial side of the cotyledon reached its final amount 4–5 d after germination, while the number of stomata on the abaxial side increased for up to 7 d after germination (Figs 9, 10).

**Fig. 11.**
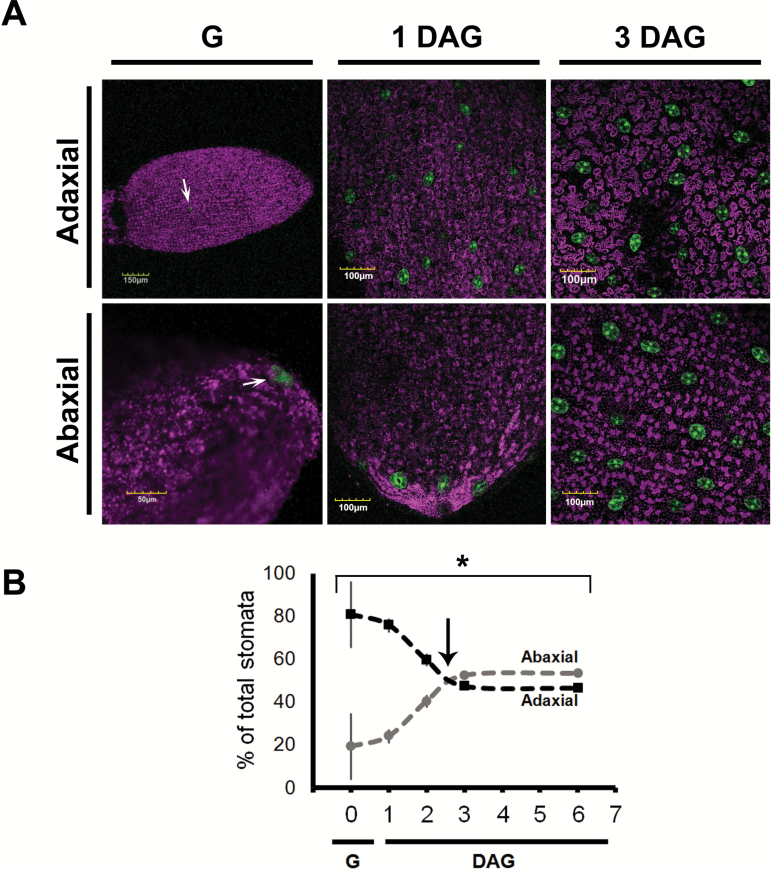
Guard cell formation in tobacco GCGFP seedlings through 6 days after germination (DAG). (A) The upper column corresponds to the adaxial side of the cotyledons and the lower column corresponds to the abaxial side of the cotyledons. The row on the left corresponds to the germination stage immediately after the removal of the seed coat. The middle row corresponds to 1 DAG, and the row on the right corresponds to 3 DAG. The white arrows point to the first stomata on the abaxial and adaxial sides of the leaf. All panels are merged images of chlorophyll autofluorescence (stained magenta) and GFP fluorescence (stained green). (B) The spatio-temporal distribution of stomata between the abaxial and adaxial sides of tobacco cotyledons. The total number of stomata (abaxial+adaxial sides of a cotyledon) was set to 100% and the distribution of stomata on the adaxial versus abaxial side was documented up to 6 DAG. Black arrows indicate the estimated inversion point, between 2 and 3 DAG. The total number of stomata was averaged each day for the adaxial (black dashed line) or abaxial (gray dashed line) sides of at least two cotyledons of three independent seedlings ±SE. When not visible, the SE is smaller than the symbol. An asterisk denotes a significant difference (*t*-test, *P*<0.05). G, germination.

Tobacco seeds were sown on 1/2 MS agar plates and then transferred to a growth chamber at 25 °C with a 16 h light/8 h dark photoperiod. As in Arabidopsis, germination was defined as the time at which the seedling penetrated the seed coat and occurred ~5 d after the transfer to the growth chamber. The number of stomata on the adaxial side of the leaf was greater than the number of stomata on the abaxial side through the first 2 d after germination (Fig. 11). On the adaxial side, the first stoma appeared in the middle of the cotyledon (at the germination stage, only a few of the seedlings developed stomata) and, on the abaxial side, the first stoma appeared at the tip of the cotyledon. That pattern was observed during the first day after germination (Fig. 11A). During the first 3 d after germination, more stomata were formed on the adaxial side than on the abaxial side. The final stomatal distribution 6 d after germination was 53.5% on the abaxial side and 46.5% on the adaxial side (Fig. 11B).

## Discussion

Most of the previous studies of guard cell-specific promoters involved the *Solanaceae* and *Brassicaceae* families (Supplementary Table S1). This study reports that *KST1*_ppro_ acts as a type I guard cell-specific promoter in tobacco, cucumber, grape, and developed barley leaves, in addition to the previously described expression in guard cells of Arabidopsis, tomato, potato, and citrus. *KST1*_ppro_ also acts a type II dominant-preferred promoter in developed citrus leaves and in young barley leaves, in which the promoter also drives expression in few epidermis cells. We therefore believe that *KST1*_ppro_ has the potential to be a good tool for controlling guard cell gene expression in various species including cucumber and barley.

*KST1*_ppro_ is also active under stress conditions such as drought and ABA treatment, and only moderately reduced expression was observed under drought conditions, as might be expected for an *StKST1* gene that encodes a potassium transporter required for stomatal opening (Fig. 6). It has been reported that the full-length and minimal promoters of *AtMYB60* are also expressed in guard cells and are repressed by drought and ABA (Rusconi *et al.*, 2013). The positive responses of the *rd29A* promoter to dehydration and ABA, which were in addition to those of the *MYB60* promoter, abolished the negative effect of those treatments and allowed stable expression (Rusconi *et al.*, 2013). The expression of *KST1*_ppro_ under drought and ABA treatment indicates that this promoter is sufficient to drive expression in guard cells under various environmental conditions.

Previous studies have demonstrated that the Dof-binding site motif (T/A)AAAG may contribute to guard cell specificity and that the three consecutive (T/A)AAAG motifs found in the *KST1* promoter (236 bp upstream of the ATG) are essential for guard cell-specific gene expression (Cominelli *et al.*, 2011; Plesch *et al.*, 2001). The sequence of *KST1*_ppro_ includes four (T/A)AAAG motifs on the 5'–3' strand and another three on the 3'–5' stand (Supplementary Fig. S4). Yet, a detailed study of promoter sequences for guard cell-expressed genes and those of globally expressed genes showed no preference with regard to the number of (T/A)AAAAG motifs, questioning the correlation between guard cell specificity and the number of (T/A)AAAG motifs (Yang *et al.*, 2008).

### Type II promoters and co-ordinated expression in guard cells and vascular tissues

Anatomical studies have pointed to parallel co-ordinated development of stomatal density and vein density that may determine overall transpiration (recently reviewed by Brodribb *et al.*, 2016). Interestingly, during the search for guard cell promoters, it was noted that in addition to guard cells, a large group of type II promoters are also active in vascular tissues (*ROP11*, *ROPGEF4*, *GORK*, *MYB61, RHC1*, *PHO1*, *TGG1*, *Myr1Bn1*, *KAT1*, *KAT2*, *TRE1*, *CYP707A1*, *KEA1*, *KEA2*, *KEA3*, *SAV6*, *AO1*, *OsKAT2*, and *SIRK*; Supplementary Table S1). This observation raises the possibility that apart from development, a synchronized response can occur at the physiological level as well, where upon the same signal, promoters are activated simultaneously in both tissues: stomata and vasculature. The rationale for such a co-ordinated response is that whole-plant water transport depends on the ongoing balance between the hydraulic activity of roots, stem, and leaves, together with guard cell aperture size adjustments (Sack and Holbrook, 2006). Therefore, such a parallel response may be required to balance water transport with water loss, to avoid embolism and cavitation.

ABA is an example of a signal that triggers such an effect, regulating water loss by stimulating stomatal closure and regulating hydraulic conductance in the bundle sheath of vascular tissues (Parent *et al.*, 2009; Shatil-Cohen *et al.*, 2011; Pantin *et al.*, 2013). The fact that a large group of guard cell promoters are also expressed in vascular tissues raises the question of whether additional signals, other than ABA, might also trigger similar parallel responses in guard cells and vascular tissues. It is likely that such signals are present in both tissues. Following this logic, a possible candidate for such a signal is sucrose, which has been suggested to induce stomatal closure (Outlaw, 2003; Kang *et al.*, 2007; Kelly *et al.*, 2013; Lawson *et al.*, 2014; Daloso *et al.*, 2016) and is the main sugar transported by the phloem in most crop plants. As it is loaded into and unloaded out of the vascular tissues, sucrose may act as a signal, triggering the activation of promoters simultaneously in the guard cells and the vascular tissue. This common stomatal–vasculature co-ordinated response is still speculative and requires extensive study.

### KST1*_ppro_*::GFP *and the distribution of stomata in germinating seedlings*

*KST1*_ppro_ is expressed immediately after the symmetric division of a GMC and that expression intensifies as stomata develop (Fig. 7A). This finding is in line with a previous study that found that the *KST1*_ppro_ Arabidopsis homolog *KAT1* has a similar expression pattern (Lai *et al.*, 2005). Since both *AtKAT1* and *StKST1* encode a potassium channel required for the uptake of potassium ions necessary for stomatal opening, it might indeed be expected that the *KAT1* and *KST1*_ppro_ promoters will be active only once a guard cell pair has been formed, as we observed here (Fig. 7A).

### Temporal and spatial formation and distribution of stomata

It appears that *KST*_ppro_ is expressed in newly formed guard cells immediately after GMC division, allowing us to follow the temporal and spatial distribution of stomata from the early stages of germination. In Arabidopsis and tobacco, the stomata are formed first primarily on the adaxial sides of the cotyledons that face each other. It is possible that the main advantage of such behavior is to avoid pathogen infiltration and mechanical damage as the seedlings make their way through the soil. We refer to this behavior as ‘protected development’. The rapid formation of stomata on the adaxial side probably ensures an immediate capability for gas exchange once the seedling reaches the light and the cotyledons unfold. The cotyledons’ need for an immediate capability to absorb CO_2_ is further supported by the fact that the first true leaves appear 4 d after germination (Fig. 9B1) and the stomata of true leaves are first seen 5 d after germination, on the distal tip of the newly developed leaves (Fig. 9C, C2).

We believe that our method of using a *KST1*_ppro_*::GFP* might have an advantage for monitoring newly formed stomata. One major advantage of this method is the simultaneous and easy detection of guard cells on both sides of the same cotyledons and of the two cotyledons of the same seedling. Once guard cells are formed, *KST1*_ppro_ drives constitutive expression (Fig. 7) that can be used to study the spatio-temporal distribution of stomata throughout development (Figs 8–11). In a previous study, it was reported that stomata are formed on the adaxial side of the cotyledons ~12 h after the formation of stomata on the abaxial side (Geisler and Sack, 2002). The difference between the results obtained by Geisler and Sack (2002) and those presented here may stem from different experimental conditions (e.g. 12 h light, in their study as compared with 16 h light in our study) or from the different methodologies used. In the previous study, the authors extrapolated data to the entire area of the cotyledon from the mean of several sampled fields. Such extrapolation may exclude certain areas of the cotyledon, particularly during the early stages (e.g. in Fig. 8A2). When using the *KST1*_ppr_::GFP method, the entire cotyledon area is imaged at the early stages of seedling germination and development (from germination through 2 d after germination) and, therefore, the collected data include all of the stomata in the cotyledon, thus reducing the chance of excluding stomata. In our study, extrapolation from specific fields to the entire leaf area started from 3 d after germination, as the cotyledons became too big for a complete image.

Unlike cotyledons, stomata are seen only on the upper part of the hypocotyl and only 2 d after germination (Fig. 8C, C1), when the hypocotyl is most probably above ground, also perhaps to avoid physical or pathogen damage. In a previous study, Berger *et al.* (1998) showed that stomata appear on the hypocotyls of Arabidopsis Landsberg ecotype (L*er*) 4 d after germination. Yet, in the Col-0 ecotype, stomata appear on the hypocotyls 2 d after germination, in agreement with our results (Kono *et al.*, 2007). In summary, among the many guard cell promoters listed in Supplementary Table S1 we believe that *KST1*_ppro_ has the potential to be useful in following guard cell appearance and function in a wide range of species and under various growing conditions.

## Supplementary data

Supplementary data are available at *JXB* online.

Fig. S1. GFP expression under the control of *KST1*_ppro_ in additional transgenic lines.

Fig. S2. GFP expression under the control of *KST1*_ppro_ is specific to guard cells in Arabidopsis and tomato.

Fig. S3. GFP under the control of *KST1*_ppro_ is preferentially expressed in the guard cells of citrus.

Fig. S4. The sequence of *KST1*_ppro_.

Table S1. List of guard cell-specific and guard cell-preferred promoters.

Table S2. Quantitative real-time PCR primers used in this study.

Video S1. Confocal microscopy 3-D movie providing a 360° tour within the leaf including the epidermis and mesophyll of potato, tobacco, cucumber, grapevine, mature barley, Arabidopsis, tomato, and young citrus.

## Supplementary Material

supplementary_figures_S1_S4_Tables_S1_S2

supplementary_video_S1
