# Supplementary material for: The Solanum tuberosum KST1 partial promoter as a tool for guard cell expression in multiple plant species
Source: J Exp Bot. 2017 May 22;68(11):2885–97. doi: 10.1093/jxb/erx159 (PMC5853950; doi:10.1093/jxb/erx159)
Supplement: supplementary_figures_S1_S4_Tables_S1_S2 [file erx159_suppl_supplementary_figures_s1_s4_tables_s1_s2.pdf]

Supplementary data

The Solanum tuberosum KST1 partial promoter as a tool for guard cell expression in multiple plant species

Gilor Kelly, Nitsan Lugassi, Eduard Belausov, Dalia Wolf, Belal Khamaisi, Danja Brandsma, Jayaram Kottapalli, Lena Fidel, Bat-Sheva Ben-Zvi, Aiman Egbaria, Atiako Kwame Acheampong, Chuanlin Zheng, Etti Or, Assaf Distelfeld, Rakefet David-Schwartz, Nir Carmi, and David Granot

Fig. S1

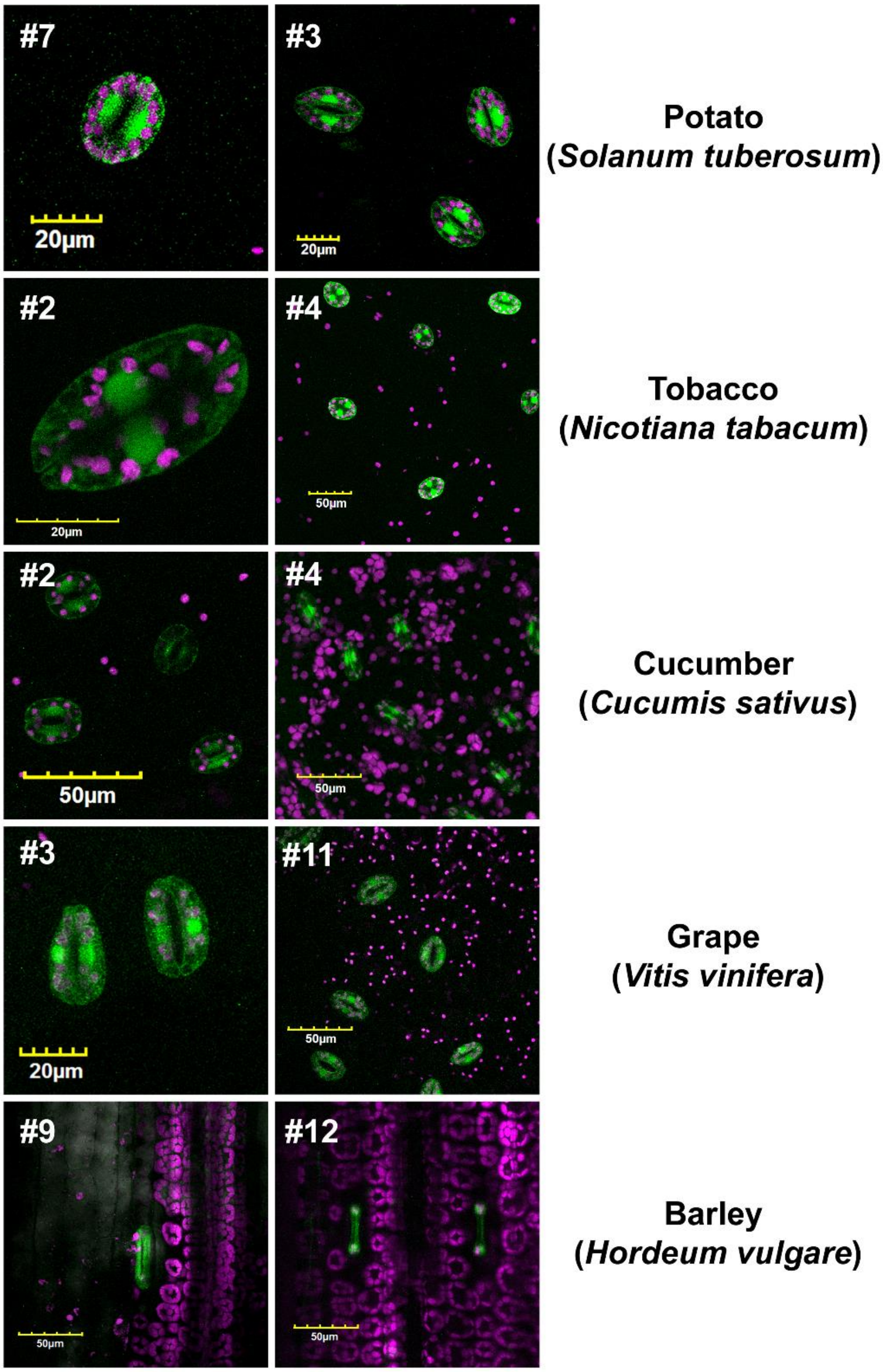

Fig. S1. GFP expression under the control of $KST1_{ppro}$ in additional transgenic lines. Confocal images of two additional transgenic lines of the newly introduced GCGFP species used in this study: potato, tobacco, cucumber, grape and barley. All panels are merged images of chlorophyll autofluorescence (stained magenta) and GFP fluorescence (stained green) taken from mature leaves. The same overall expression pattern was observed for all lines tested in this study (3 to 10 independent lines for each species). Scale bars (yellow) are defined in each image.

Fig. S2

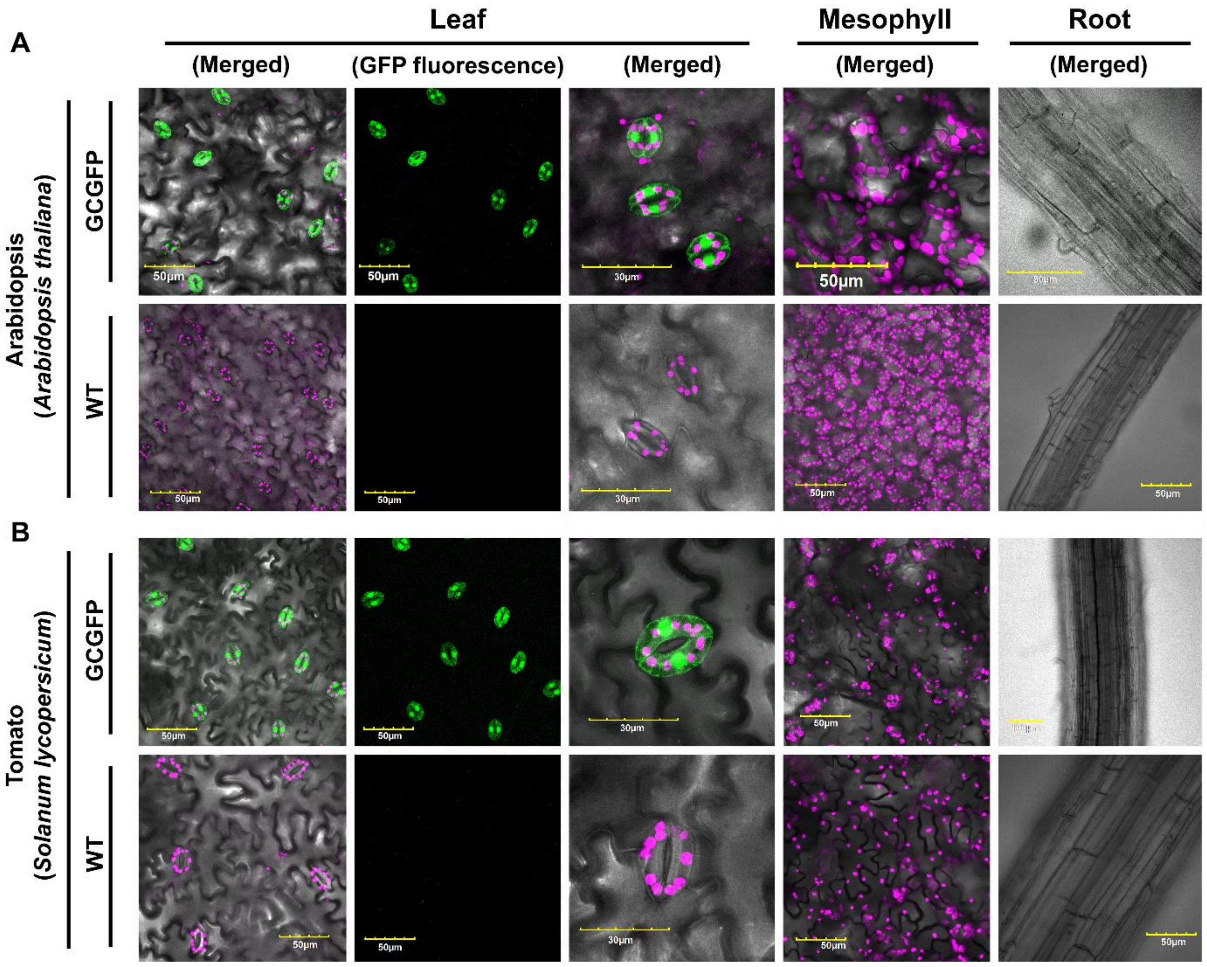

Fig. S2. GFP expression under the control of $\mathit{KST1}_{\mathrm{ppro}}$ is specific to guard cells in Arabidopsis (A) and tomato (B). (A–B) Confocal images of leaf, mesophyll and root of wild-type (WT) and transgenic plants expressing GFP under the control of $\mathit{KST1}_{\mathrm{ppro}}$ (GCGFP). Unless mentioned otherwise, all panels are merged images of white light, chlorophyll autofluorescence (stained magenta) and GFP fluorescence (stained green). Scale bars (yellow) are defined in each image.

Fig. S3

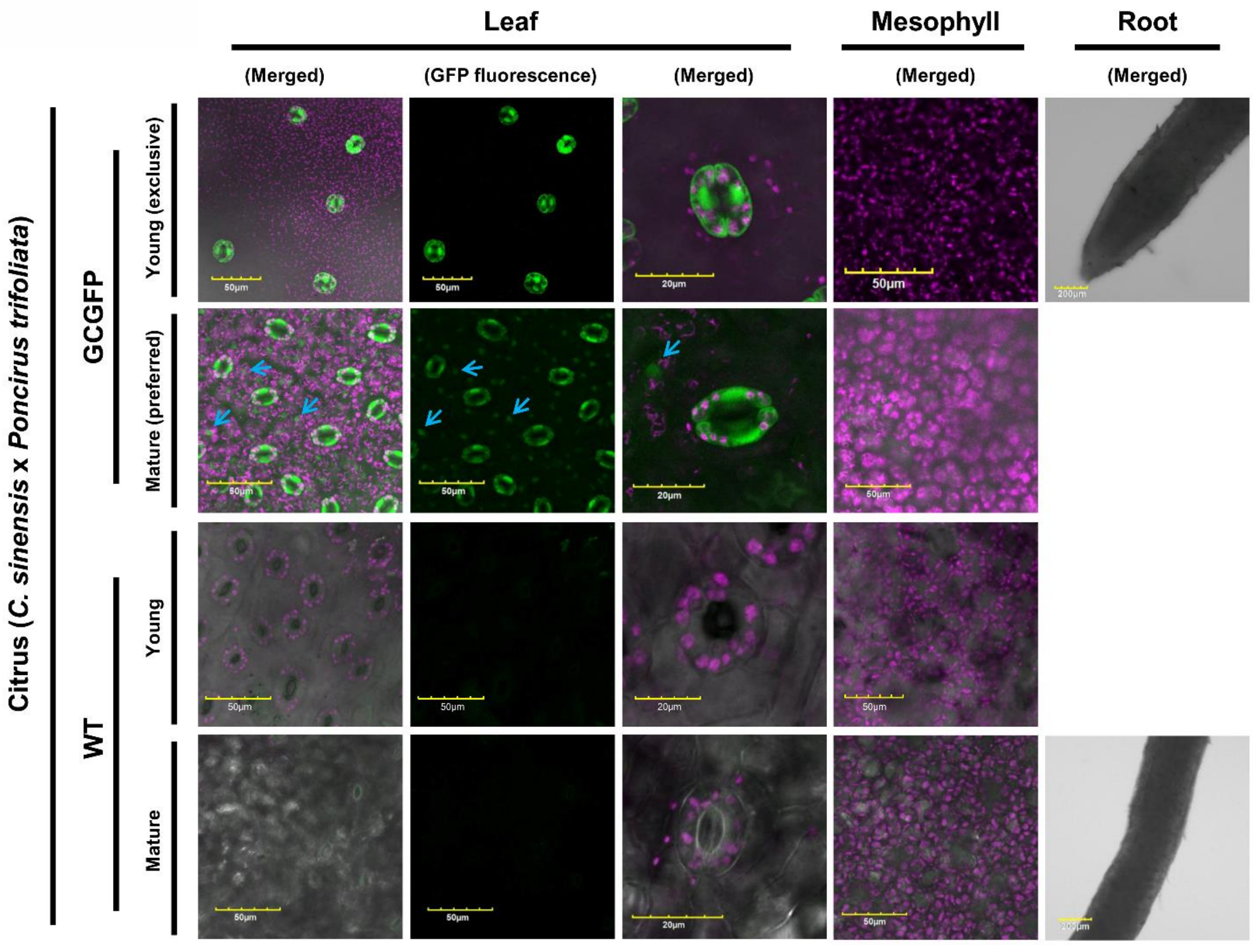

Fig. S3. GFP under the control of *KST1*$_{ppro}$ is preferentially expressed in the guard cells of citrus. Confocal images of young and mature leaves (about two months old), mesophyll and roots of wild-type (WT) and transgenic plants expressing GFP under the control of the *KST1*$_{ppro}$ (GCGFP). Unless mentioned otherwise, all panels are merged images of white light, chlorophyll autofluorescence (stained magenta) and GFP fluorescence (stained green). Blue arrows indicate the location of epidermal cells. Scale bars (yellow) are defined in each image.

Fig. S4

***KST1*$_{ppro}$ promoter sequence**

1 CTAGAAAATGAAATGAAAAACACCTATCTGTTTTTTCACTCAAATTCCATCCTTGCAATA

61 AAATGCTTATTCTTAAAATTTCTATCTTGGTGGAGATCCCACCACCATTACCATTTTCCC

121 CAAAAATCTTACAACATTATTTCCATTTTCTTTCTCTTAATTCTCTCAACAAATTCCCCT

181 TGCACCTGAATTATTATCAAAGAAAATCATGTTTGCCACTTCAACAACTTTATAACTCAT

241 ATCTCGCCTCGTTTGTTGTTTATTGTGTTTCAATATTGCTATGTTTTCTTCTATTTTGTA

301 CTTGCATTTGCTCACTCGAGCTTTTGGTAACAATCTCTCTACTTCTACTAGATCTGCGTA

361 CAGTCTACCTTCTCCAGACCCCACTTGTGGGAAGATACTATAGAAGTAGGCAAGTAGCAA

421 TGTCACGTTCT**TAAAG**CTAAATGCTTTTTC**AAAAG**AATCACAA**TAAAG**AAACACTTGACC

481 CGTGTATCACCCCAACTACTTCTTCATCTACATCCTCTATATATAAACACGCTAAAAATA

541 ACTAGTTAGTATTTTTAAATATTACACATTGCCTTTCCAAGAAACTCGAAAAAAAAAAAA

601 AAAAAAAAAAAAACCACATCAACAA**AAAAG**AAGCAGCAATATATAATA

Fig. S4. The sequence of *KST1*$_{ppro}$. (T/A)AAAG motifs on the 5’-3’ strand are in bold and on the 3’-5’ stand are underlined.

Table S1

Table S1. List of guard cell-specific and guard cell-preferred promoters. ***Type I***, guard cell-specific promoters: expression was not detected in vegetative tissues other than guard cells. ***Type II***, guard cell-preferred promoters: dominant expression in guard cells with expression also detected in non-stomatal vegetative tissues. Data include promoters that were shown to be active in guard cells using reporter genes. [1] Promoters that are active during stomatal development, as well as in the guard cells of mature stomata (contradictory findings are also noted). [2] Promoters that were tested mostly in cotyledons and for which there is insufficient data to determine the promoter type at later stages of plant development. Enhancer trap lines used to isolate guard-cell promoters are also included in the table. GC, guard cell; GFP, green fluorescence protein; YFP, yellow fluorescence protein; GUS, β-glucoronidase; YC3.60, GFP-based calcium reporter yellow cameleon 3.60; Citrine, citrine fluorescent protein; DsRed2, Discosoma sp. Red fluorescent protein 2; ABA, abscisic acid.

Promoter	Gene accession no.	Species examined<br>(+) expressed<br>(-) not expressed	Verification method	Type:<br>Guard cell-specific (I) or preferred (II) promoters	Expression in vegetative tissues other than guard cells	Comments	References
***EXPA1* (*Arabidopsis thaliana*)**	AT1G69530	Arabidopsis (+)	GUS	I	-		(Zhang *et al.*, 2011)
***MYB60* (*Arabidopsis thaliana*)**	AT1G08810	Arabidopsis (+)<br>tobacco (+)<br>tomato (+)<br>rice (-)	GUS, GFP	I	-	Expression was also detected in anthers of tomato and tobacco (Rusconi *et al.*, 2013)	(Cominelli *et al.*, 2011; Cominelli *et al.*, 2005; Galbiati *et al.*, 2008; Meyer *et al.*, 2010; Nagy *et al.*, 2009; Oh *et al.*, 2011; Rusconi *et al.*, 2013)
***CYP86A2* (*Arabidopsis thaliana*)**	AT4G00360	Arabidopsis (+)	GFP	I	-		(Francia *et al.*, 2008)
***GC1* (*Arabidopsis thaliana*)**	AT1G22690	Arabidopsis (+)<br>tobacco (+)	GUS,<br>YC3.60,<br>GFP	I	-		(Kinoshita *et al.*, 2011; Na *et al.*, 2015; Wang *et al.*, 2014; Yang *et al.*, 2008)

***SLAC1* (*Arabidopsis thaliana*)**	AT1G12480	Arabidopsis (+)	GUS	I		Expression was sometimes observed in vascular tissue and was also detected in stamens and siliques.	(Imai *et al.*, 2015; Negi *et al.*, 2008; Vahisalu *et al.*, 2008)
***RAB18* (*Arabidopsis thaliana*)**	AT5G66400	Arabidopsis (+)	GFP	I	-		(Merilo *et al.*, 2015; Waadt *et al.*, 2014)
***gcPEPC* (*Solanum tuberosum*)**	AY210895	Arabidopsis (+)	Citrine, DsRed2	I	-		(Kopka *et al.*, 1997; Muller-Rober *et al.*, 1998; Xiong *et al.*, 2009)
***SKT1* (*Solanum tuberosum*)**	AF237951	potato (+)	GUS	I	-	Active primarily on the abaxial leaf surface	(Zimmermann *et al.*, 2001)
***AGPase* partial promoter (*Solanum tuberosum*)**	X75017 (promoter seq.)	potato (+) tobacco (+) Arabidopsis (+)	GUS	I	-		(Berger and Altmann, 2000; Muller-Rober *et al.*, 1994)
***KST1* full promoter**	X79779	potato (+)	GUS	I	-	Expression was also detected in flowers.	(Muller-Rober *et al.*, 1998; Muller-Rober *et al.*, 1995;

**(*Solanum tuberosum*)**							Plesch *et al.*, 2001)
***KST1*-chimeric *p*4XKST82bp Promoter (*Solanum tuberosum*)**	X79779	potato (+) tobacco (+)	GFP, GUS	I	-		(Na and Metzger, 2014)
***KST1* partial promoter (*Solanum tuberosum*)**	X79779	potato (+) Arabidopsis (+) tomato (+) citrus (+) tobacco (+) cucumber (+) grapevine (+) barley (+)	GUS, GFP	I - potato, Arabidopsis, tomato, tobacco, cucumber, grapevine II - citrus, barley	Epidermis of young barley leaves and mature citrus leaves		(Kelly *et al.*, 2013; Lugassi *et al.*, 2015; Plesch *et al.*, 2001; Sade *et al.*, 2014; current manuscript)
SLSP* partial promoter *(Gossypium barbadense)	-	Arabidopsis (+) tobacco (+)	GUS, GFP	I	-	Expression was also detected in flowers.	(Han *et al.*, 2013)
***MYB60* (*Vitis vinifera*)**	ACF21938	Arabidopsis (+)	GUS	I	-	Expression was also detected in flowers (carpels and stigmas).	(Galbiati *et al.*, 2011)

SIRK **(*Vitis vinifera*)**	AF359521	grapevine (+) Arabidopsis (+)	GUS	I - grapevine II - Arabidopsis	Xylem of Arabidopsis		(Pratelli *et al.*, 2002)
DPG1 **synthetic promoter**	-	tobacco (+)	GUS	I	-		(Li *et al.*, 2005)
ASPG1 **(*Arabidopsis thaliana*)**	AT3G18490	Arabidopsis (+)	GUS	I	Not specified	Analysis included only aerial tissues	(Yao *et al.*, 2012)
CHX20 **(*Arabidopsis thaliana*)**	AT3G53720	Arabidopsis (+)	GUS	II	Root cap		(Padmanaban *et al.*, 2007)
ROP11 **(*Arabidopsis thaliana*)**	AT5G62880	Arabidopsis (+)	GUS, GFP	II	Germinating seeds, veins of cotyledons, vascular tissues of roots and developed leaves	Expression was also detected in pollen.	(Li *et al.*, 2012)
ROPGEF4 **(*Arabidopsis***	AT2G45890	Arabidopsis (+)	GUS	II	Roots, vascular tissues	Expression was also detected in pollen.	(Li and Liu, 2012)

***thaliana*)**							
***GORK* (*Arabidopsis thaliana*)**	AT5G37500	Arabidopsis (+)	GFP (transient), GUS	II	Roots, vascular system, root hairs		(Becker *et al.*, 2003)
***MYB61* (*Arabidopsis thaliana*)**	AT1G09540	Arabidopsis (+)	GUS, GFP	II	Vascular tissue of young seedling, roots	Expression was also detected in inflorescence stem and seeds	(Liang *et al.*, 2005; Newman *et al.*, 2004; Penfield *et al.*, 2001)
***RHC1* (*Arabidopsis thaliana*)**	AT4G22790	Arabidopsis (+)	GUS	II	Vascular tissues	Expression was also detected in flowers.	(Tian *et al.*, 2015)
***PAO3* (*Arabidopsis thaliana*)**	AT3G59050	Arabidopsis (+)	GUS	II	Roots, hypocotyls, stipules and trichomes	Expression was also detected in pollen.	(Fincato *et al.*, 2012)
***PHO1* (*Arabidopsis thaliana*)**	AT3G23430	Arabidopsis (+)	GUS	II	vascular tissue	Induced by ABA treatment	(Zimmerli *et al.*, 2012)
ALMT12* (*Arabidopsis	AT4G17970	Arabidopsis (+)	GUS	II	Steles of roots	Expression was also detected in pollen.	(Meyer *et al.*, 2010; Sasaki *et al.*, 2010)

***thaliana*)**							
RHA1 **(*Arabidopsis thaliana*)**	AT5G45130	Arabidopsis (+)	GUS	II	Seeds, hypocotyl, stipules and roots	Expression was also detected in receptacle	(Terryn *et al.*, 1993)
CER6 **(*Arabidopsis thaliana*)**	AT1G68530	Arabidopsis (+) tobacco (+)	GUS, GFP	II	Epidermis		(Hooker *et al.*, 2002; Kinoshita *et al.*, 2011; Tsuzuki *et al.*, 2013)
TGG1 **(*Arabidopsis thaliana*)**	AT5G26000	Arabidopsis (+) tobacco (+)	GUS	II	Distinct expression in the phloem		(Husebye *et al.*, 2002; Thangstad *et al.*, 2004)
Myr1.Bn1 **(*Brassica napus)***	AF323020	Arabidopsis (+) *B. napus* (+)	GUS	II	Distinct expression in the phloem		(Thangstad *et al.*, 2004)
SCAP1 **(*Arabidopsis thaliana*)**	AT5G65590	Arabidopsis (+)	GUS, GFP	II	Not specified	Authors reported preferred expression.	(Negi *et al.*, 2013)
KAT1 **(*Arabidopsis thaliana*)**	AT5G46240 (gene),	Arabidopsis (+)	GUS	II	Vascular tissue of roots	GUS staining in roots was observed in only 2 of the 11 lines tested.	(Lai *et al.*, 2005; Nakamura *et al.*, 1995)

	U25088 (promoter + gene seq.)						
***KAT2* (*Arabidopsis thaliana*)**	AT4G18290 (AJ288900)	Arabidopsis (+)	GUS	II	Phloem of minor veins in developed leaves, non-specific expression in developing leaves		(Kinoshita *et al.*, 2011; Pilot *et al.*, 2001)
***MPK12* (*Arabidopsis thaliana*)**	AT2G46070	Arabidopsis (+)	GUS, GFP	II	Non-specific expression in seedlings,<br><br>low expression in roots		(Jammes *et al.*, 2009; Lee *et al.*, 2009)
***TRE1* (*Arabidopsis thaliana*)**	AT4G24040	Arabidopsis (+)	GUS	II	Leaf veins, germinating seeds, basal part of hypocotyls, hydathodes of young leaves, apical meristerm	Expression was also detected in flowers.	(Van Houtte *et al.*, 2013)
***CYP707A1* (*Arabidopsis thaliana*)**	AT4G19230	Arabidopsis (+)	GUS	II	Vascular tissue	Expressed under high humidity	(Okamoto *et al.*, 2009)
***KEA1* (*Arabidopsis thaliana*)**	AT1G01790	Arabidopsis (+)	GUS	II	Vascular tissue, hypocotyl	Expression was also detected in flowers.	(Han *et al.*, 2015)

***KEA2* (*Arabidopsis thaliana*)**	AT4G00630	Arabidopsis (+)	GUS	II	Roots, vascular tissue, hypocotyl	Expression was also detected in flowers.	(Han *et al.*, 2015)
***KEA3* (*Arabidopsis thaliana*)**	AT4G04850	Arabidopsis (+)	GUS	II	Vascular tissue, hypocotyl	Expression was also detected in flowers.	(Han *et al.*, 2015)
***KEA4* (*Arabidopsis thaliana*)**	AT2G19600	Arabidopsis (+)	GUS	II	Roots and trichomes	Expression was also detected in flowers.	(Han *et al.*, 2015)
***KEA5* (*Arabidopsis thaliana*)**	AT5G51710	Arabidopsis (+)	GUS	II	Roots and trichomes	Expression was also detected in flowers.	(Han *et al.*, 2015)
***SAV6* (*Arabidopsis thaliana*)**	AT5G26680	Arabidopsis (+)	GUS	II	Trichomes, meristems, vascular tissues and endodermal cells of hypocotyls and roots	Expression was also detected in flowers (carpels and stigmas).	(Zhang *et al.*, 2015)
***AO1* (*Arabidopsis thaliana*)**	AT4G14940	Arabidopsis (+)	GUS, GFP	II	Xylem		(Ghuge *et al.*, 2015a, b)

GTL1 **(Arabidopsis thaliana)**	AT1G33240	Arabidopsis (+)	GUS,GFP	II	Epidermis		(Yoo *et al.*, 2010)
NHX2 **(*Arabidopsis thaliana*)**	AT3G05030	Arabidopsis (+)	GUS	II	Non-specific in seedlings, expressed around vasculature of roots and meristems	Expression was also detected in flowers.	(Barragán *et al.*, 2012)
PDR12*/ *ABCG40 **(*Arabidopsis thaliana*)**	AT1G15520	Arabidopsis (+)	GUS	II	Non-specific expression in young plantlets, expression in roots		(Kang *et al.*, 2010)
RNaseLER **(*Solanum lycopersicum*)**	AM408589	Tobacco (+)	GUS	II	Trichomes and the parenchyma of stem cells	Expression was also detected in flowers.	(Kothke and Kock, 2011)
KAT2 **(*Oryza sativa*)**	OS01G11250	Rice (+)	GUS	II	Weak expression in vascular tissues		(Hwang *et al.*, 2013a; Hwang *et al.*, 2013b)
***ADS* partial promoter** **(*Artemisia annua*)**	DQ448294	*A. annua* (-) Arabidopsis (+)	GUS	II	Trichomes		(Zhu *et al.*, 2014)

CDeT6-19 **(*Craterostigma plantagineum*)**	X74067	Arabidopsis (+) tobacco (+)	GUS	II	Epidermal cells adjacent to stomatal complex	Active in response to ABA and drought stress	(Michel *et al.*, 1993; Taylor *et al.*, 1995)
**Promoters active during stomatal development and in mature guard cells**							
SPCH [1] **(*Arabidopsis thaliana*)**	AT5G53210	Arabidopsis (+)	GUS, GFP	I	-	Active in mature stomata of cotyledons, hypocotyls and rosette as well as at earlier stages of stomatal development; inactive in mature stomata according to some reports	(Hunt and Gray, 2009; Lee *et al.*, 2014; MacAlister *et al.*, 2007; Pillitteri *et al.*, 2007)
MUTE [1,2] **(*Arabidopsis thaliana*)**	AT3G06120	Arabidopsis (+)	GUS, GFP	I	-	Active in mature stomata of cotyledons and at earlier stages of stomatal development; inactive in mature stomata according to some reports	(Hunt and Gray, 2009; Lee *et al.*, 2014; Pillitteri *et al.*, 2008; Yamamuro *et al.*, 2014)
ERL1 [1,2] **(*Arabidopsis***	AT5G62230	Arabidopsis (+)	GUS	I	-	Active in mature stomata of cotyledons and at	(Pillitteri *et al.*, 2007; Shpak *et al.*, 2005)

***thaliana*)**						earlier stages of stomatal development	
ERL2 [1,2] **(*Arabidopsis thaliana*)**	AT5G07180	Arabidopsis (+)	GUS	I	-	Weak expression; active in mature stomata of cotyledons and at earlier stages of stomatal development	(Shpak *et al.*, 2005)
EPF1 [1,2] **(*Arabidopsis thaliana*)**	AT2G20875	Arabidopsis (+)	GFP, GUS, YFP	I	-	Active in mature stomata of cotyledons and at earlier stages of stomatal development	(Hara *et al.*, 2007, 2009; Hunt and Gray, 2009; Matos *et al.*, 2014)
FAMA [1,2] **(*Arabidopsis thaliana*)**	AT3G24140	Arabidopsis (+)	GUS, GFP	I	-	Weak expression; active in mature stomata of cotyledons and at earlier stages of stomatal development	(Lee *et al.*, 2014; Ohashi-Ito and Bergmann, 2006; Pillitteri *et al.*, 2007; Vanneste *et al.*, 2011)
SCREAM/ICE 1 [1,2] **(*Arabidopsis thaliana*)**	AT3G26744	Arabidopsis (+)	GUS	I	-	Weak expression; active in mature stomata of cotyledons and at earlier stages of stomatal development	(Kanaoka *et al.*, 2008)
CDKB1;1 [1,2] **(*Arabidopsis thaliana*)**	AT3G54180	Arabidopsis (+)	GUS	I	-	Active in mature stomata of cotyledon and at earlier stages of stomatal development	(Boudolf *et al.*, 2004)

**Enhancer trap lines**						
**E1728 enhancer trap line (*Arabidopsis thaliana*)**	-	Arabidopsis (+)	GFP, GUS	II	Myrosin cells (Shirakawa *et al.*, 2014)	(Akita *et al.*, 2013; Azoulay-Shemer *et al.*, 2015; Dodd *et al.*, 2006; Gardner *et al.*, 2009; Higaki *et al.*, 2014; Meng and Yao, 2015; Ohashi-Ito and Bergmann, 2006; Shirakawa *et al.*, 2014; Yang *et al.*, 2014)
**E361-1 enhancer trap line (*Arabidopsis thaliana*)**	-	Arabidopsis (+)	GFP	II	Epidermal pavement cells and roots	(Gardner *et al.*, 2009; Qian *et al.*, 2013)
**KS019-1 enhancer trap line (*Arabidopsis thaliana*)**	-	Arabidopsis (+)	GFP	II	Leaf apical meristem, leaf primordia and root epidermis	(Gardner *et al.*, 2009)
**J2103-1 enhancer trap line (*Arabidopsis thaliana*)**	-	Arabidopsis (+)	GFP	II	Epidermal pavement cells on the surface of hypocotyls and the adaxial leaf surface, as well as	(Gardner *et al.*, 2009)

	the roots (cap, tips and vascular tissue)	

References

Akita K, Hasezawa S, Higaki T. 2013. Breaking of plant stomatal one-cell-spacing rule by sugar solution immersion. PLoS One **8,** e72456.

Azoulay-Shemer T, Palomares A, Bagheri A, Israelsson-Nordstrom M, Engineer CB, Bargmann BOR, Stephan AB, Schroeder JI. 2015. Guard cell photosynthesis is critical for stomatal turgor production, yet does not directly mediate CO_2- and ABA-induced stomatal closing. The Plant Journal, **83,** 567–581.

Barragán V, Leidi EO, Andrés Z, Rubio L, De Luca A, Fernández JA, Cubero B, Pardo JM. 2012. Ion exchangers NHX1 and NHX2 mediate active potassium uptake into vacuoles to regulate cell turgor and stomatal function in Arabidopsis. The Plant Cell **24,** 1127–1142.

Becker D, Hoth S, Ache P, Wenkel S, Roelfsema MR, Meyerhoff O, Hartung W, Hedrich R. 2003. Regulation of the ABA-sensitive Arabidopsis potassium channel gene GORK in response to water stress. FEBS Letters **554,** 119–126.

Boudolf V, Barrôco R, Engler Jde A, Verkest A, Beeckman T, Naudts M, Inzé D, De Veylder L. 2004. B1-type cyclin-dependent kinases are essential for the formation of stomatal complexes in *Arabidopsis thaliana*. The Plant Cell **16,** 945–955.

Cominelli E, Galbiati M, Vavasseur A, Conti L, Sala T, Vuylsteke M, Leonhardt N, Dellaporta SL, Tonelli C. 2005. A guard-cell-specific MYB transcription factor regulates stomatal movements and plant drought tolerance. Current Biology **15,** 1196–1200.

Dodd AN, Jakobsen MK, Baker AJ, *et al.* 2006. Time of day modulates low-temperature Ca^{2+} signals in Arabidopsis. The Plant Journal **48,** 962–973.

Fincato P, Moschou PN, Ahou A, Angelini R, Roubelakis-Angelakis KA, Federico R, Tavladoraki P. 2012. The members of *Arabidopsis thaliana* PAO gene family exhibit distinct tissue- and organ-specific expression pattern during seedling growth and flower development. Amino Acids **42,** 831–841.

Francia P, Simoni L, Cominelli E, Tonelli C, Galbiati M. 2008. Gene trap-based identification of a guard cell promoter in Arabidopsis. Plant Signaling & Behavior **3,** 684–686.

Galbiati M, Simoni L, Pavesi G, Cominelli E, Francia P, Vavasseur A, Nelson T, Bevan M, Tonelli C. 2008. Gene trap lines identify Arabidopsis genes expressed in stomatal guard cells. The Plant Journal **53,** 750–762.

Gardner MJ, Baker AJ, Assie JM, Poethig RS, Haseloff JP, Webb AA. 2009. GAL4 GFP enhancer trap lines for analysis of stomatal guard cell development and gene expression. Journal of Experimental Botany **60,** 213–226.

Ghuge SA, Carucci A, Rodrigues-Pousada RA, Tisi A, Franchi S, Tavladoraki P, Angelini R, Cona A. 2015. The apoplastic copper AMINE OXIDASE1 mediates jasmonic acid-induced protoxylem differentiation in Arabidopsis roots. Plant Physiology **168,** 690–707.

Ghuge SA, Carucci A, Rodrigues-Pousada RA, Tisi A, Franchi S, Tavladoraki P, Angelini R, Cona A. 2015. The MeJA-inducible copper amine oxidase AtAO1 is expressed in xylem tissue and guard cells. Plant Signaling & Behavior **10,** e1073872.

Han L, Han YN, Xiao XG. 2013. Truncated cotton subtilase promoter directs guard cell-specific expression of foreign genes in tobacco and Arabidopsis. PLoS ONE **8,** e59802.

Han L, Li JL, Wang L, Shi WM, Su YH. 2015. Identification and localized expression of putative K^+/H^+ antiporter genes in Arabidopsis. Acta Physiologiae Plantarum **37,** 1–14.

Hara K, Kajita R, Torii KU, Bergmann DC, Kakimoto T. 2007. The secretory peptide gene EPF1 enforces the stomatal one-cell-spacing rule. Genes & Development **21,** 1720–1725.

Hara K, Yokoo T, Kajita R, Onishi T, Yahata S, Peterson KM, Torii KU, Kakimoto T. 2009. Epidermal cell density is autoregulated via a secretory peptide, EPIDERMAL PATTERNING FACTOR 2 in Arabidopsis leaves. Plant & Cell Physiology **50,** 1019–1031.

Higaki T, Kutsuna N, Hasezawa S. 2014. CARTA-based semi-automatic detection of stomatal regions on an Arabidopsis cotyledon surface. Plant Morphology **26,** 9–12.

Hunt L, Gray JE. 2009. The signaling peptide EPF2 controls asymmetric cell divisions during stomatal development. Current Biology **19,** 864–869.

Hwang H, Yoon J, Kim HY, *et al.* 2013*a*. Unique features of two potassium channels, OsKAT2 and OsKAT3, expressed in rice guard cells. PLoS ONE **8,** e72541.

Hwang H, Yoon JY, Cho H, Kim BG. 2013*b*. OsKAT2 is the prevailing functional inward rectifier potassium channels in rice guard cell. Plant Signaling & Behavior **8,** e26643.

Imai H, Noda Y, Tamaoki M. 2015. Alteration of Arabidopsis SLAC1 promoter and its association with natural variation in drought tolerance. Plant Signaling & Behavior **10,** e989761.

Kanaoka MM, Pillitteri LJ, Fujii H, Yoshida Y, Bogenschutz NL, Takabayashi J, Zhu JK, Torii KU. 2008. SCREAM/ICE1 and SCREAM2 specify three cell-state transitional steps leading to arabidopsis stomatal differentiation. The Plant Cell **20,** 1775–1785.

Kang J, Hwang JU, Lee M, Kim YY, Assmann SM, Martinoia E, Lee Y. 2010. PDR-type ABC transporter mediates cellular uptake of the phytohormone abscisic acid. Proceedings of the National Academy of Sciences, USA **107,** 2355–2360.

Kinoshita T, Ono N, Hayashi Y, *et al.* 2011. FLOWERING LOCUS T regulates stomatal opening. Current Biology **21,** 1232–1238.

Kopka J, Provart NJ, MullerRober B. 1997. Potato guard cells respond to drying soil by a complex change in the expression of genes related to carbon metabolism and turgor regulation. The Plant Journal **11,** 871–882.

Köthke S, Köck M. 2011. The *Solanum lycopersicum* RNaseLER is a class II enzyme of the RNase T2 family and shows preferential expression in guard cells. Journal of Plant Physiology **168,** 840–847.

Lee E, Lucas JR, Goodrich J, Sack FD. 2014. Arabidopsis guard cell integrity involves the epigenetic stabilization of the FLP and FAMA transcription factor genes. The Plant Journal **78,** 566–577.

Lee JS, Wang S, Sritubtim S, Chen J-G, Ellis BE. 2009. Arabidopsis mitogen-activated protein kinase MPK12 interacts with the MAPK phosphatase IBR5 and regulates auxin signaling. The Plant Journal **57,** 975–985.

Li J, Gong X, Lin H, Song Q, Chen J, Wang X. 2005. DGP1, a drought-induced guard cell-specific promoter and its function analysis in tobacco plants. Science China Life Sciences **48,** 181–186.

Li Z, Kang J, Sui N, Liu D. 2012. ROP11 GTPase is a negative regulator of multiple ABA responses in Arabidopsis. Journal of Integrative Plant Biology **54,** 169–179.

Li Z, Liu D. 2012. ROPGEF1 and ROPGEF4 are functional regulators of ROP11 GTPase in ABA-mediated stomatal closure in Arabidopsis. FEBS Letters **586,** 1253–1258.

Liang YK, Dubos C, Dodd IC, Holroyd GH, Hetherington AM, Campbell MM. 2005. AtMYB61, an R2R3-MYB transcription factor controlling stomatal aperture in *Arabidopsis thaliana*. Current Biology **15,** 1201–1206.

MacAlister CA, Ohashi-Ito K, Bergmann DC. 2007. Transcription factor control of asymmetric cell divisions that establish the stomatal lineage. Nature **445,** 537–540.

Meng X, Chen X, Mang H, Liu C, Yu X, Gao X, Torii KU, He P, Shan L. 2015. Differential function of Arabidopsis SERK family receptor-like kinases in stomatal patterning. Current Biology **25,** 2361–2372.

Merilo E, Jalakas P, Laanemets K, Mohammadi O, Hõrak H, Kollist H, Brosché M. 2015. Abscisic acid transport and homeostasis in the context of stomatal regulation. Molecular Plant **8,** 1321–1333.

Meyer S, Mumm P, Imes D, *et al*. 2010. AtALMT12 represents an R-type anion channel required for stomatal movement in Arabidopsis guard cells. The Plant Journal **63,** 1054–1062.

Michel D, Salamini F, Bartels D, Dale P, Baga M, Szalay A. 1993. Analysis of a desiccation and ABA-responsive promoter isolated from the resurrection plant *Craterostigma plantagineum*. The Plant Journal **4,** 29–40.

Na JK, Kim JK, Kim DY, Assmann SM. 2015. Expression of potato RNA-binding proteins StUBA2a/b and StUBA2c induces hypersensitive-like cell death and early leaf senescence in Arabidopsis. Journal of Experimental Botany **66,** 4023–4033.

Na JK, Metzger JD. 2014. Chimeric promoter mediates guard cell-specific gene expression in tobacco under water deficit. Biotechnology Letters **36,** 1893–1899.

Nagy R, Grob H, Weder B, Green P, Klein M, Frelet-Barrand A, Schjoerring JK, Brearley C, Martinoia E. 2009. The Arabidopsis ATP-binding cassette protein

AtMRP5/AtABCC5 is a high affinity inositol hexakisphosphate transporter involved in guard cell signaling and phytate storage. The Journal of Biological Chemistry **284,** 33614–33622.

Nakamura RL, McKendree WL Jr, Hirsch RE, Sedbrook JC, Gaber RF, Sussman MR. 1995. Expression of an Arabidopsis potassium channel gene in guard cells. Plant Physiology **109,** 371–374.

Negi J, Matsuda O, Nagasawa T, Oba Y, Takahashi H, Kawai-Yamada M, Uchimiya H, Hashimoto M, Iba K. 2008. CO_2 regulator SLAC1 and its homologues are essential for anion homeostasis in plant cells. Nature **452,** 483–486.

Negi J, Moriwaki K, Konishi M, *et al.* 2013. A Dof transcription factor, SCAP1, is essential for the development of functional stomata in Arabidopsis. Current Biology **23,** 479–484.

Newman LJ, Perazza DE, Juda L, Campbell MM. 2004. Involvement of the R2R3-MYB, AtMYB61, in the ectopic lignification and dark-photomorphogenic components of the det3 mutant phenotype. The Plant Journal **37,** 239–250.

Oh JE, Kwon Y, Kim JH, Noh H, Hong SW, Lee H. 2011. A dual role for MYB60 in stomatal regulation and root growth of *Arabidopsis thaliana* under drought stress. Plant Molecular Biology **77,** 91–103.

Ohashi-Ito K, Bergmann DC. 2006. Arabidopsis FAMA controls the final proliferation/differentiation switch during stomatal development. The Plant Cell **18,** 2493–2505.

Okamoto M, Tanaka Y, Abrams SR, Kamiya Y, Seki M, Nambara E. 2009. High humidity induces abscisic acid 8'-hydroxylase in stomata and vasculature to regulate local and systemic abscisic acid responses in Arabidopsis. Plant Physiology **149,** 825–834.

Padmanaban S, Chanroj S, Kwak JM, Li X, Ward JM, Sze H. 2007. Participation of endomembrane cation/H^+ exchanger AtCHX20 in osmoregulation of guard cells. Plant Physiology **144,** 82–93.

Penfield S, Meissner RC, Shoue DA, Carpita NC, Bevan MW. 2001. MYB61 is required for mucilage deposition and extrusion in the Arabidopsis seed coat. The Plant Cell **13,** 2777–2791.

Pillitteri LJ, Bogenschutz NL, Torii KU. 2008. The bHLH protein, MUTE, controls differentiation of stomata and the hydathode pore in Arabidopsis. Plant & Cell Physiology **49,** 934–943.

Pillitteri LJ, Sloan DB, Bogenschutz NL, Torii KU. 2007. Termination of asymmetric cell division and differentiation of stomata. Nature **445,** 501–505.

Qian P, Han B, Forestier E, Hu Z, Gao N, Lu W, Schaller H, Li J, Hou S. 2013. Sterols are required for cell-fate commitment and maintenance of the stomatal lineage in Arabidopsis. The Plant Journal **74,** 1029–1044.

Sasaki T, Mori IC, Furuichi T, Munemasa S, Toyooka K, Matsuoka K, Murata Y, Yamamoto Y. 2010. Closing plant stomata requires a homolog of an aluminum-activated malate transporter. Plant & Cell Physiology **51,** 354–365.

Shirakawa M, Ueda H, Nagano AJ, Shimada T, Kohchi T, Hara-Nishimura I. 2014. FAMA is an essential component for the differentiation of two distinct cell types, myrosin cells and guard cells, in Arabidopsis. The Plant Cell **26,** 4039–4052.

Shpak ED, McAbee JM, Pillitteri LJ, Torii KU. 2005. Stomatal patterning and differentiation by synergistic interactions of receptor kinases. Science **309,** 290–293.

Taylor JE, Renwick KF, Webb AA, *et al*. 1995. ABA-regulated promoter activity in stomatal guard cells. The Plant Journal **7,** 129–134.

Terryn N, Arias MB, Engler G, Tiré C, Villarroel R, Van Montagu M, Inzé D. 1993. rha1, a gene encoding a small GTP binding protein from Arabidopsis, is expressed primarily in developing guard cells. The Plant Cell **5,** 1761–1769.

Thangstad OP, Gilde B, Chadchawan S, Seem M, Husebye H, Bradley D, Bones AM. 2004. Cell specific, cross-species expression of myrosinases in *Brassica napus*, *Arabidopsis thaliana* and *Nicotiana tabacum*. Plant Molecular Biology **54,** 597–611.

Tian W, Hou C, Ren Z, *et al.* 2015. A molecular pathway for CO_2 response in Arabidopsis guard cells. Nature Communications **6,** 6057.

Tsuzuki T, Takahashi K, Tomiyama M, Inoue S, Kinoshita T. 2013. Overexpression of the Mg-chelatase H subunit in guard cells confers drought tolerance via promotion of stomatal closure in *Arabidopsis thaliana*. Frontiers in Plant Science **4,** 440.

Vahisalu T, Kollist H, Wang YF, *et al.* 2008. SLAC1 is required for plant guard cell S-type anion channel function in stomatal signalling. Nature **452,** 487–491.

Van Houtte H, Vandesteene L, López-Galvis L, *et al.* 2013. Overexpression of the trehalase gene AtTRE1 leads to increased drought stress tolerance in Arabidopsis and is involved in abscisic acid-induced stomatal closure. Plant Physiology **161,** 1158–1171.

Waadt R, Hitomi K, Nishimura N, Hitomi C, Adams SR, Getzoff ED, Schroeder JI. 2014. FRET-based reporters for the direct visualization of abscisic acid concentration changes and distribution in Arabidopsis. eLife **3,** e01739.

Wang Y, Hills A, Blatt MR. 2014. Systems analysis of guard cell membrane transport for enhanced stomatal dynamics and water use efficiency. Plant Physiology **164,** 1593–1599.

Xiong TC, Hann CM, Chambers JP, Surget M, Ng CK. 2009. An inducible, modular system for spatio-temporal control of gene expression in stomatal guard cells. Journal of Experimental Botany **60,** 4129–4136.

Yamamuro C, Miki D, Zheng Z, Ma J, Wang J, Yang Z, Dong J, Zhu JK. 2014. Overproduction of stomatal lineage cells in Arabidopsis mutants defective in active DNA demethylation. Nature Communications **5,** 4062.

Yang K, Wang H, Xue S, Qu X, Zou J, Le J. 2014. Requirement for A-type cyclin-dependent kinase and cyclins for the terminal division in the stomatal lineage of Arabidopsis. Journal of Experimental Botany **65,** 2449–2461.

Yoo CY, Pence HE, Jin JB, Miura K, Gosney MJ, Hasegawa PM, Mickelbart MV. 2010. The Arabidopsis GTL1 transcription factor regulates water use efficiency and drought tolerance by modulating stomatal density via transrepression of SDD1. The Plant Cell **22,** 4128–4141.

Zhang XQ, Wei PC, Xiong YM, Yang Y, Chen J, Wang XC. 2011. Overexpression of the Arabidopsis α-expansin gene AtEXPA1 accelerates stomatal opening by decreasing the volumetric elastic modulus. Plant Cell Reports **30,** 27–36.

Zhang Y, Wen C, Liu S, Zheng L, Shen B, Tao Y. 2016. Shade avoidance 6 encodes an Arabidopsis flap endonuclease required for maintenance of genome integrity and development. Nucleic Acids Research **44,** 1271–1284.

Zhu MM, Zhang FY, Lv ZY, *et al*. 2014. Characterization of the promoter of artemisia annua amorpha-4,11-diene synthase (ADS) gene using homologous and heterologous expression as well as deletion analysis. Plant Molecular Biology Reporter **32,** 406–418.

Zimmerli C, Ribot C, Vavasseur A, Bauer H, Hedrich R, Poirier Y. 2012. PHO1 expression in guard cells mediates the stomatal response to abscisic acid in Arabidopsis. The Plant Journal **72,** 199–211.

Zimmermann S, Hartje S, Ehrhardt T, Plesch G, Mueller-Roeber B. 2001. The K^+ channel SKT1 is co-expressed with KST1 in potato guard cells—both channels can co-assemble via their conserved KT domains. The Plant Journal **28,** 517–527.

Table S2

Table S2. Quantitative real-time PCR primers used in this study

Gene name	Primer sequence
***AtRAB 18*_F**	TTACCAGAACCGTCCAGGAG
***AtRAB 18*_R**	ACCACCACCAGTTCCGTATC
***AtTUB2*_F**	AAACTCACTACCCCCAGCTTTG
***AtTUB2*_R**	CACCAGACATAGTAGCAGAAATCAAGT
***GFP*_F**	AGAACGGCATCAAGGTGAAC
***GFP*_R**	TGCTCAGGTAGTGGTTGTCG
***SlCyP*_F**	CGTCGTGTTTGGACAAGTTG
***SlCyP*_R**	CCGCAGTCAGCAATAACCA
***SlNCED1*_F**	GGTGAACCGAAACAAACTCG
***SlNCED1*_R**	ACCATCGTCTTCTTCCTTGC

Movie S1

Movie S1. Confocal microscopy 3-D movie providing a 360° tour within the leaf including the epidermis and mesophyll of potato, tobacco, cucumber, grapevine, mature barley, Arabidopsis, tomato, and young citrus. The movie images were color-coded green for GFP and magenta for chlorophyll autofluorescence. Bars (white line) are specified for each species.
